# Acrylamide alters CREB and retinoic acid signalling pathways during differentiation of the human neuroblastoma SH-SY5Y cell line

**DOI:** 10.1038/s41598-020-73698-6

**Published:** 2020-10-07

**Authors:** Kristina Attoff, Ylva Johansson, Andrea Cediel-Ulloa, Jessica Lundqvist, Rajinder Gupta, Florian Caiment, Anda Gliga, Anna Forsby

**Affiliations:** 1 Department of Biochemistry and Biophysics, Stockholm University, Stockholm, Sweden; 2 Unit of Toxicology Sciences, Swedish Toxicology Sciences Research Center (Swetox), Karolinska Institutet, Södertälje, Sweden; 3 Department for organismal biology, Uppsala University, Uppsala, Sweden; 4 Institute of Environmental Medicine, Karolinska Institutet, Stockholm, Sweden; 5 Department of Toxicogenomics, School of Oncology and Developmental Biology (GROW), Maastricht University, Maastricht, The Netherlands

**Keywords:** Cell biology, Developmental biology, Genetics, Molecular biology, Neuroscience, Risk factors

## Abstract

Acrylamide (ACR) is a known neurotoxicant which crosses the blood–brain barrier, passes the placenta and has been detected in breast milk. Hence, early-life exposure to ACR could lead to developmental neurotoxicity. The aim of this study was to elucidate if non-cytotoxic concentrations of ACR alter neuronal differentiation by studying gene expression of markers significant for neurodevelopment in the human neuroblastoma SH-SY5Y cell model. Firstly, by using RNASeq we identified two relevant pathways that are activated during 9 days of retinoic acid (RA) induced differentiation i.e. RA receptor (RAR) activation and the cAMP response element-binding protein (CREB) signalling pathways. Next, by qPCR we showed that 1 and 70 µM ACR after 9 days exposure alter the expression of 13 out of 36 genes in the RAR activation pathway and 18 out of 47 in the CREB signalling pathway. Furthermore, the expression of established neuronal markers *i.e. BDNF, STXBP2*, *STX3*, *TGFB1* and *CHAT* were down-regulated. Decreased protein expression of BDNF and altered ratio of phosphorylated CREB to total CREB were confirmed by western blot. Our results reveal that micromolar concentrations of ACR sustain proliferation, decrease neurite outgrowth and interfere with signalling pathways involved in neuronal differentiation in the SH-SY5Y cell model.

## Introduction

Acrylamide (ACR) can be generated from food components during heat treatment as a result of the Maillard reaction between amino acids and reducing sugars or reactive carbonyls^1^. Most people are exposed to ACR through food intake but there is also a risk of continuous exposure from environmental pollutants since polymerized ACR is used in for example water management and in cosmetic products^2,3^. ACR is easily absorbed and distributed throughout the body, with the nervous system being a particularly susceptible target organ^4^. ACR also crosses the placenta and has been detected in breast milk, which results in exposure of the foetus and the infant child^5,6^. ACR is currently classified as a probable carcinogen (Group 2A)^2^ and has been shown to induce neurotoxicity, reproductive toxicity, genotoxicity and carcinogenicity^7,8^. Some studies show that ACR may induce developmental neurotoxicity (DNT). Prenatal and perinatal ACR exposure affect average horizontal motor activity, auditory startle response and cerebellar development rats^9,10^, learning and memory function are impaired in young mice that have been exposed with low doses of ACR^11^, prenatal ACR exposure in rats induces lipid peroxidation and oxidative stress^9^ and impairs development of hippocampal neurons in weaning rats^12^. In vitro studies have shown that ACR interferes with neuronal differentiation and alters transcriptional markers for neurodevelopment in the C17.2 neural progenitor cell model^13^, attenuates neurite outgrowth in PC12 cells^14^, delays maturation of primary neurons^11^ and impairs synaptic function^15^.

The developing central nervous system (CNS) is more susceptible to chemicals and adverse effects can be observed at lower doses compared to the mature CNS^16,17^. Even though the foetus is partially protected by the placenta, some substances can cross the placental barrier due to their physicochemical properties such as their lipid solubility, protein binding, molecular weight or their degree of ionization^18^. The blood–brain barrier and the blood-cerebrospinal fluid barrier are completely developed at approximately 6 months post-partum, which could allow toxicants to reach the brain of foetuses and new-borns in the first months of life^19,20^. The causalities for DNT can be very complex since many different developmental processes may be affected including proliferation, migration, differentiation, synaptogenesis and apoptosis of cells^21^. The processes are regulated by gene expression in a controlled and timely manner at critical periods of development. Alterations of these processes could lead to detrimental effects in the offspring^22^.

All-trans retinoic acid (RA) is a regulator of neurodevelopment and an inducer of neuronal differentiation in cell models. Alterations to RA receptor (RAR) activation pathway have been associated with severe adverse consequences to the developing nervous system^23,24^. The cAMP response element-binding protein (CREB) is a transcription factor and one of the most important regulators of the brain-derived neurotrophic factor (BDNF)^25^. Neuronal proliferation, plasticity, survival and differentiation are correlated with CREB activation^26,27^. ACR has been shown to affect RA-induced differentiation in vitro^28–30^ and to interfere with CREB signalling in vivo^31^. Therefore, our hypothesis was that exposure with low concentrations of ACR during neuronal differentiation may alter gene expression of markers for neuronal differentiation and signalling, including the RAR and CREB signalling pathways and thus, attenuate neurite outgrowth and sustain proliferation, i.e. cellular hallmarks of DNT.

Here, we evaluated effects on DNT-associated markers in the human neuroblastoma SH-SY5Y cell model after ACR exposure with concentrations that were non-cytotoxic according to the cytotoxicity assays while still affecting neuronal differentiation by reducing the number of neurites per cell and neurite length. Using a transcriptomics approach, we first explored the differentially expressed genes (DEGs) and enriched pathways during 9 days of RA induced differentiation of SH-SY5Y cells. Two of these pathways, i.e. RAR activation and CREB signalling in neurons, together with well-established markers for neuronal differentiation and function were selected for further evaluation by quantitative RT-PCR (qPCR). Effects of ACR on the expression of BDNF, CREB and ratio of phosphorylated CREB (pCREB) in relation to total CREB were also confirmed at the protein level, after exposure with concentrations that had a clear effect on differentiation in terms of sustained proliferation and impaired neurite outgrowth, but no effect on cell viability.

## Results

### Transcriptomics analysis of differentiating SH-SY5Y cells reveals robust time-dependent changes in gene expression

**Figure 1:**
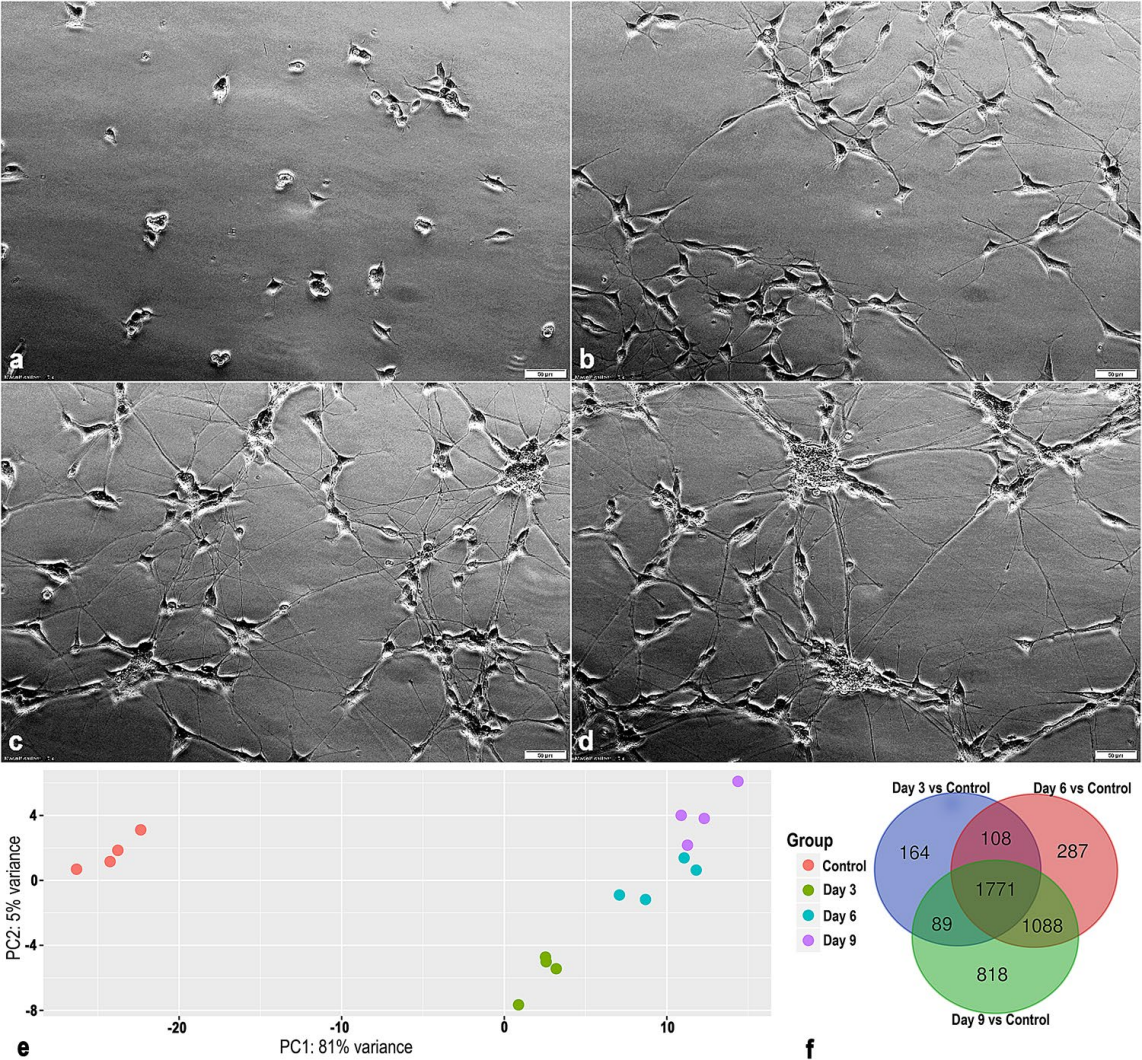
Phase contrast images of RA-induced differentiation of SH-SY5Y cells after **(a)** 0 (Control), **(b)** 3, **(c)** 6 and **(d)** 9 days. Scale bar = 50 µm. **(e)** Changes in gene expression during 3, 6 and 9 days of differentiation of the SH-SY5Y cells. Principal component analysis plot of rlog transformed count data for the RNA-sequencing samples of 4 individual experiments (N = 4). Each replicate clusters according to their individual differentiation status. **(f)** Venn diagram showing overlap of differentially expressed genes between the different time points of differentiation of SH-SY5Y cells. Genes with FDR-adjusted* p*-value ≤ 0.05 and an absolute log2(fold change) > 1 were regarded as differentially expressed. Unprocessed phase contrast images are represented in Supplementary Figure S6.

The SH-SY5Y cells displayed a clear differentiated morphological phenotype after treatment with RA (Fig. 1a–d). Principal component analysis reveals that the RNA-sequencing data clusters according to the differentiation status (Fig. 1e). The first two principal components explained 86% of the information (variation) of the dataset (the variance for PC1 being 81% and 5% for PC2). Genes that had an absolute log2(fold change) expression > 1 together with a false discovery rate (FDR)-adjusted* p*-value ≤ 0.05 were defined as differentially expressed and selected for further analysis. The Venn diagram (Fig. 1f) shows the number of differentially expressed genes (DEGs) that overlap between the different contrasts, i.e. undifferentiated cells, 3, 6 and 9 days of differentiation. We identified 2132 DEGs after three days (1735 upregulated and 397 downregulated), 3254 DEGs after 6 days (2303 upregulated and 951 downregulated) and 3767 DEGs after 9 days (2572 upregulated and 1196 downregulated) of differentiation in comparison to the undifferentiated cells. There were 1771 DEGs that overlapped between the three contrasts.

#### Pathway analysis gives insight into the biological changes during neural differentiation in the SH-SY5Y cell model

Ingenuity pathway analysis (IPA) software was used to perform canonical pathway analysis for the DEGs after 9 days of differentiation. Table 1 contains the enriched canonical pathways after 9 days of differentiation. The selection was based on significant pathways that were part of the ‘Neurotransmitters and other nervous system signalling’ and ‘Ingenuity toxicity pathways’ categories. The top pathways according to the level of significance were the Axonal Guidance pathway followed by the Hepatic Fibrosis/Hepatic Stellate Cell Activation pathway. The Axonal Guidance pathway includes important processes for axonal extension, which is crucial for the formation of a functional nervous system^32^. The “Hepatic Fibrosis pathway” is defined by genes related to the extracellular matrix and not by liver specific genes, and is indicative of matrix remodelling during neuronal differentiation^33^. We decided to focus on two of the enriched pathways, i.e. the RAR activation (Supplementary Fig. S1) and the CREB signalling in neurons (Supplementary Fig. S2) for further evaluation after exposure to ACR. These pathways were carefully selected due to their known importance for neuronal development and their implications in DNT. It has also been shown that ACR alters RA-induced differentiation in vitro^28–30^ and interferes with CREB signalling in vivo^31^. Nevertheless, 19 of the 47 genes included in the CREB signalling pathway (21 of the, in total, 90 genes selected for qPCR analysis after ACR exposure) were also included in the most significant enriched pathway, i.e. the Axonal Guidance Signalling canonical pathway. Furthermore, 21 of all markers were included in the Dopamine-DARPP32 feedback in cAMP signalling pathway and 12 markers in the GABA receptor signalling pathway. Twelve markers were identical in the CREB and Glutamate receptor signalling pathways. Hence, the selection of the 92 markers for further analysis after ACR exposure during differentiation is representative for not only the RA and CREB signalling pathways, but also for several significant enriched neuronal pathways identified in the SH-SY5Y cells (Supplementary Table S1).

**Table 1:** The enriched canonical pathways identified by DEGs after 9 days of differentiation. The selection was based on the significant pathways part of the ‘Neurotransmitters and other nervous system signalling’ and ‘Ingenuity toxicity pathways’ categories according to the IPA software.

Ingenuity canonical pathways	−log(*p*-value)	z-score
Axonal guidance signalling	8.24	NA
Hepatic fibrosis/hepatic stellate cell activation	5.29	NA
Synaptic long term depression	4.09	3.62
Cell cycle: G1/S checkpoint regulation	3.99	0.94
p53 Signalling	3.92	0.58
Cell cycle: G2/M DNA damage checkpoint regulation	3.85	1.00
Neuropathic pain signalling in dorsal horn neurons	3.72	3.41
GABA receptor signalling	3.51	NA
Dopamine-DARPP32 feedback in cAMP signalling	3.43	2.47
GPCR-mediated nutrient sensing in enteroendocrine cells	3.35	4.02
CREB signalling in neurons	3.07	2.96
CDK5 signalling	3.00	2.29
Aryl hydrocarbon receptor signalling	2.69	-1.40
Reelin signalling in neurons	2.66	NA
Opioid signalling pathway	2.50	3.39
Circadian rhythm signalling	2.20	NA
Glutamate receptor signalling	2.01	0.33
Agrin interactions at neuromuscular junction	1.98	3.15
Netrin signalling	1.98	NA
GNRH signalling	1.90	3.00
Parkinson's signalling	1.84	NA
RAR activation	1.45	NA
ErbB2-ErbB3 signalling	1.40	1.60
Gustation pathway	1.39	NA
NRF2-mediated oxidative stress response	1.38	1.00
Synaptic long term potentiation	1.38	2.60

z-score is a measure of the predicted activation state of the pathway. Z-score > 0 pathway predicted to be activated; z-score < 0 pathway predicted to be inhibited. NA- activity pattern not available.

The RAR signalling pathway was enriched after 9 days of differentiation, but the activity pattern was not available. The RAR signalling pathway generated in the IPA software is illustrated in Supplementary Fig. S1. The heatmap of the DEGs in the RAR signalling pathway over time in the differentiating SH-SY5Y cells is shown in Fig. 3. After 9 days, the RAR activation pathway was defined by 36 DEGs, out of which 23 (64%) were upregulated. These genes are part of several complexes such as the main signal transducers of the receptors for the transforming growth factor beta (TGF-β) superfamily (SMAD) complex (*SMAD6, SMAD7, SMAD9*) and the SWItch/Sucrose Non-Fermentable (SWI-SNF) complex family of chromatin remodelling complexes (*DPF1, PBRM1, SMARCA4*) as well as groups such as the retinol-binding protein (RBP) group (*CRABP1, CRABP2, RBP1, RBP3, RBP7*), the adenylyl cyclase (AC) group (*ADCY4, ADCY5, ADCY8*), the protein kinase C (PKC) group (*PRKCA, PRKCD, PRKCG*), the retinaldehyde dehydrogenase (ALDH) group (*ALDH1A2, ALDH1A3*), the retinoic acid receptor group (*RARB*) and the retinol dehydrogenase (RDH) group (*DHRS3, RDH16*). Seven genes overlap between the two pathways, i.e. *ADCY4/5/8, PIK3CD, PRKCA, PRKCD* and *PRKCG*.

The CREB signalling in neurons had a positive activity pattern and thus was predicted to be activated in SH-SY5Y cells after 9 days of differentiation (Table 1). The CREB signalling in neurons pathway is illustrated in Supplementary Fig. S2. The heatmap of the DEGs in the CREB signalling pathway over time in the differentiating SH-SY5Y cells is shown in Fig. 4. After 9 days of differentiation the CREB pathway was defined by 47 DEGs, out of which 38 (81%) were upregulated. These genes are part of several complexes such as the phosphoinositide 3-kinase (PI3K) complex (*FGFR1, FGFR2, FGFR4, IRS1, PIK3CD, TLR9*) and the voltage-dependent calcium channel (CACN) complex (*CACNA1B, CACNA1D, CACNA1E, CACNA1G, CACNA2D2, CACNG1, CACNG4, CACNG5, CACNG7*) as well as groups such as the phospholipase C (PLC) group (*NOTUM, PLCB2, PLCD1, PLCD3, PLCH1, PLCH2*), the G protein γ (Gγ) group (*GNG2, GNG3, GNG7*), the ionotropic glutamate receptor (iGLUR) group (*GRIA2, GRID1, GRIK4, GRIN2C, GRIN2D*), the metabotropic glutamate receptor (mGLUR) II/III group (*GRM2, GRM4, GRM7, GRM8*), the AC group (*ADCY4, ADCY5, ADCY8*) and the CREB group (*CREB3L3, CREB5*). Most of the DEGs at day 9 were differentially expressed already at day 3 and day 6. When differentially expressed the direction of the genes was consistent over time.

### Low concentrations of acrylamide attenuated differentiation without affecting cell viability

**Figure 2:**
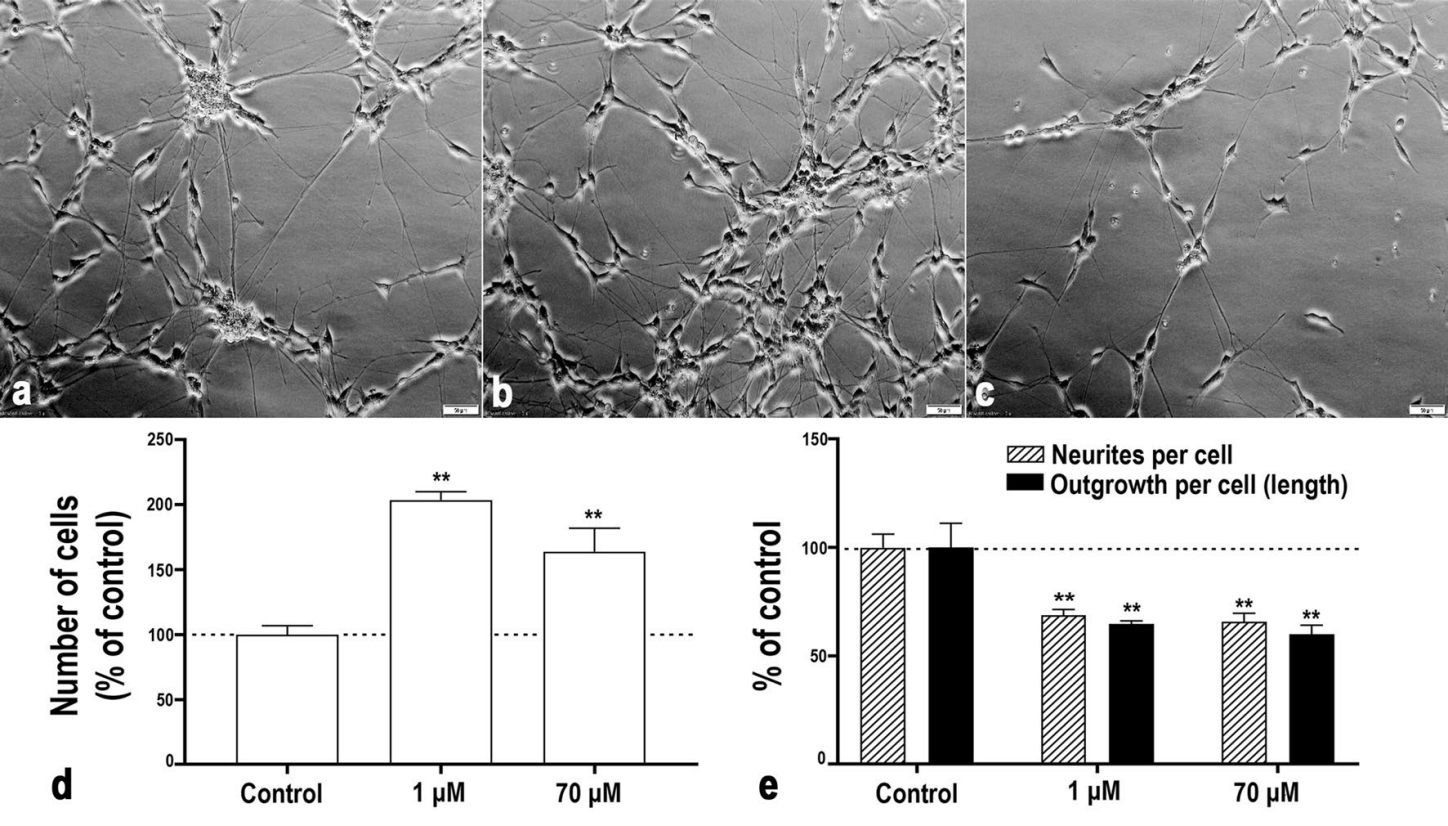
Non-cytotoxic exposure to ACR during differentiation increases the number of cells and decreases the number of nerites and neurite length in SH-SY5Y cells as compared to unexposed differentiated cells. Phase contrast images show SH-SY5Y cells exposed to ACR (**(a)** control, **(b)** 1 μM and **(c)** 70 μM) during 9 days of differentiation. Scale bar = 50 µm. **(d)** The number of cells after 9 days of differentiation and exposure to 1 or 70 μM of ACR. Results are expressed as % relative to untreated cells. **(e)** The number of neurites per cell and total neurite outgrowth (measured in length) after 9 days of differentiation and exposure to 1 or 70 μM of ACR. The data are presented as the mean of 3 independent experiments (N = 3). Results were analysed using one-way ANOVA followed by Tukey’s multiple comparisons test. Bars represent the mean ± SEM. **p ≤ 0.01 compared to control (cells exposed to medium without ACR). Unprocessed phase contrast images are represented in Supplementary Figure S7.

We first investigated the amount of viable cells and cell viability after 9 days of exposure to a very wide range of concentrations of ACR during differentiation by using the resazurin assay, the ATP content assay and resistance to propidium iodide (PI) incorporation (Supplementary Fig. S3). In the resazurin assay, ACR concentration as low as 10^−15^ M significantly increased the fluorescence intensity in exposed cells compared to control. Increased number of viable cells was also observed in the ATP assay and previously confirmed by [^3^H]thymidine incorporation in ACR exposed SH-SY5Y cells during differentiation^28^. Cells exposed to 10^−4^ M ACR or higher produced significantly less fluorescence intensity compared with control in the resazurin assay, indicating either cell death, attenuated proliferation or decreased metabolic activity. There was, however, no decrease in PI-resistant cells observed at 100 µM or lower concentrations, indicating absence of cell death. From the three different viability assays, 1 μM and 70 μM were chosen as non-cytotoxic concentrations of ACR for further investigation on effects of ACR on neuronal markers, the RAR activation and CREB signalling in neurons pathway selected at the transcriptional level, as well as the expression of BDNF, CREB and pCREB at the protein level. Increased proliferation due to attenuated differentiation can result in an imbalance in the number of neurons. To confirm that the chosen non-cytotoxic concentrations of ACR induced adversity that can be related to developmental neurotoxicity, we determined the number of cells, the number of neurites and the total neurite length after 9 days of exposure during differentiation (Fig. 2). In concurrence with the increased fluorescence witnessed in the resazurin assay and luminescence in the ATP assay, the number of cells was significantly higher and the number of neurites per cell as well as the total amount of neurite outgrowth (measured in length) were significantly lower after exposure to both 1 µM and 70 µM of ACR in comparison with unexposed control cells.

### Acrylamide interferes with RAR signalling during neuronal differentiation

**Figure 3:**
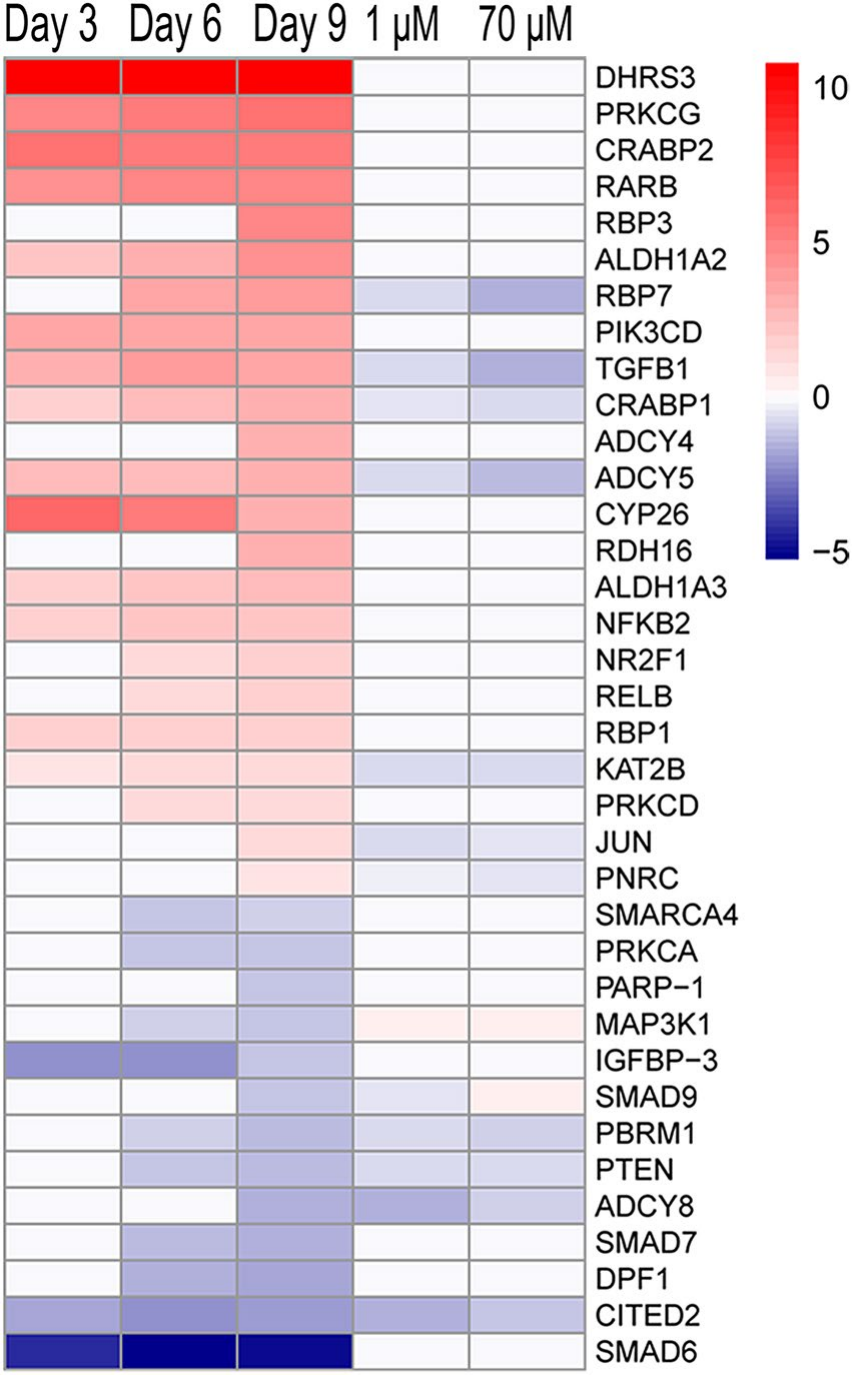
ACR interferes with the RA receptor signalling pathway during differentiation of SH-SY5Y cells. Heatmap of the gene expression changes (padj ≤ 0.05) of genes part of the RA receptor signalling pathway at different differentiation time points (Day 3, Day 6 and Day 9 versus undifferentiated cells —RNA-sequencing input data) and following exposure to ACR (1 and 70 µM, during 9 days of differentiation versus untreated cells at the same time point—qPCR input data). Colour coding refers to the log2(fold change). Genes are ranked according to decreasing log2(fold change) of the contrast Day 9 versus undifferentiated cells.

Acrylamide (1 μM and 70 μM) significantly changed the expression of 13 genes in the RAR activation pathway (Fig. 3). Differentially expressed genes denoted by the gene complexes and groups are as follows: RBP group—*CRABP1, CRABP2, RBP1, RBP3, RBP7*; PIK3K p110 group—*PIK3CD*; AC group—*ADCY4, ADCY5, ADCY8*; PKC group—*PRKCA, PRKCD, PRKCG*; ALDH group—*ALDH1A2, ALDH1A3*; RAR group—*RARB*; RDH group—*DHRS3, RDH16*; Nuclear receptor coactivator (NCOA) group -*CITED2*; Proto-oncogene REL group—*RELB*; Nuclear factor kappa-light-chain-enhancer of activated B cells (NF-kB) group—*NFkB2*; COUP transcription factor group—*NR2F1*; SMAD complex—*SMAD6, SMAD7, SMAD9*; SWI-SNF complex—*DPF1, PBRM1, SMARCA4*; TGFβ group—*TGFβ1*. The expression of the genes *RBP7, TGFB1, CRABP1, ADCY5, KAT2B, JUN* and *PNRC* were significantly decreased after exposure to ACR. The expression of these genes was all differentially increased compared to undifferentiated cells during differentiation. The genes *PBRM1, PTEN, ADCY8* and *CITED2* were also significantly decreased in their expression after ACR exposure but differentially reduced compared to undifferentiated cells during differentiation. *MAP3K1* expression was decreased compared to undifferentiated cells during differentiation and further significantly decreased after exposure to 1 and 70 μM ACR. There was one gene that showed different response to the two concentrations of ACR. The *SMAD9* expression was significantly decreased after 1 μM but significantly increased after 70 μM of ACR exposure compared to unexposed control cells. The expression of *SMAD9* was reduced during differentiation compared to undifferentiated cells.

### Acrylamide interferes with CREB signalling during neuronal differentiation

**Figure 4:**
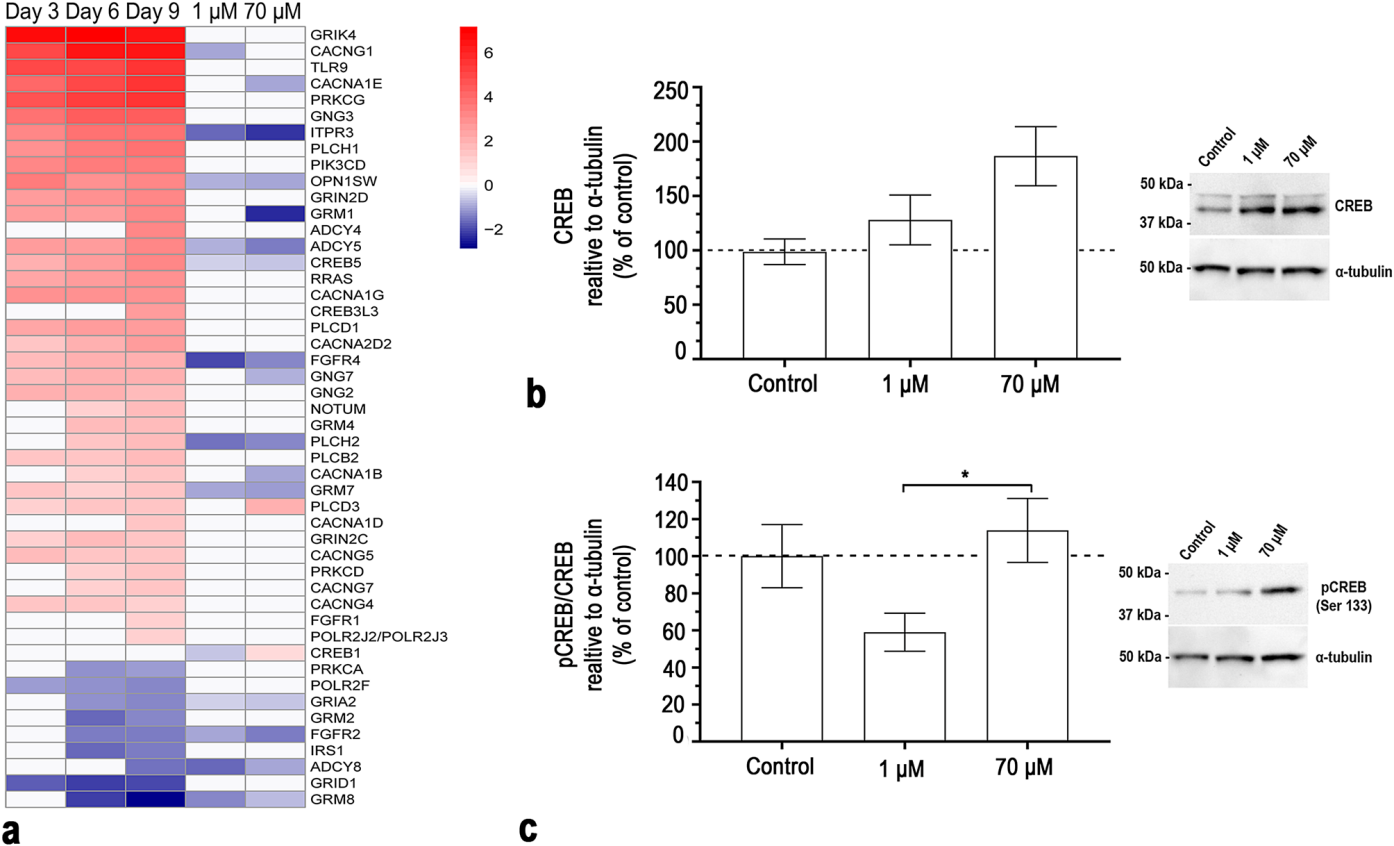
ACR interferes with the CREB signalling pathway during differentiation of SH-SY5Y cells. **(a)** Heatmap of the gene expression changes (padj ≤ 0.05) of genes part of the CREB pathway at different differentiation time points (Day 3, Day 6 and Day 9 versus undifferentiated cells —RNA-sequencing input data) and following exposure to ACR (1 and 70 µM, during 9 days of differentiation versus untreated cells at the same time point—qPCR input data). Colour coding refers to the log2(fold change). Genes are ranked according to decreasing log2(fold change) of the contrast Day 9 versus undifferentiated cells. **(b)** CREB protein levels determined after 9 days of differentiation and exposure to 1 and 70 μM of ACR by western blot (images for representative blots are included). **(c)** pCREB/CREB protein levels determined after 9 days of differentiation and exposure to 1 and 70 μM of ACR by western blot (images for representative gels are included). The data from the protein levels **(c**,**d)** were normalized against α-tubulin and presented as the mean ± SEM (N = 7). Results were analysed using one-way ANOVA followed by Dunnett’s multiple comparisons test. *p ≤ 0.05. Full length blots are represented in Supplementary Figure S4.

In the CREB signalling pathway the expression of 17 genes that were significantly changed after exposure to 1 or 70 μM of ACR (Fig. 4a). Differentially expressed genes denoted by the gene complexes and groups were as follows: Small GTPase RAS group—*RRAS*; PI3K complex—*FGFR1, FGFR2, FGFR4, IRS1, PIK3CD, TLR9*; PKC group—*PRKCA, PRKCD, PRKCG*; CaCn complex—*CACNA1B, CACNA1D, CACNA1E, CACNA1G, CACNA2D2, CACNG1, CACNG4, CACNG5, CACNG7*; PLC group—*NOTUM, PLCB2, PLCD1, PLCD3, PLCH1, PLCH2*; Inositol trisphosphate receptor (IP3R) group—*ITPR3*; Gγ group—*GNG2, GNG3, GN7*; mGLUR I group—*GRM1*; iGLUR group—*GRIA2, GRID1, GRIK4, GRIN2C, GRIN2D*: mGLUR II/III group—*GRM2, GRM4, GRM7, GRM8*; Heterotrimeric G protein alpha subunit (Gαi) group—*OPN1SW*; AC group—*ADCY4, ADCY5, ADCY8*; CREB group—*CREB3L3, CREB5*; RNA Pol II complex—*POLR2F, POLR2J2*. The expression of nine of the genes were downregulated after exposure with 1 µM as well as 70 µM of ACR. The gene expression of *ITPR3, OPN1SW, ADCY5, CREB5, PLCH2* and *GRM7* was significantly reduced and these genes were all differentially upregulated during differentiation over time compared to undifferentiated cells. *GRIA2, FGFR2* and *ADCY8* expression was also significantly reduced after exposure to ACR, however these genes were differentially reduced compared to undifferentiated cells after 9 days of differentiation. *FGFR4* expression was significantly increased after exposure to 1 μM of ACR and was differentially increased compared to undifferentiated cells during differentiation. Expression of *GRM8* was also significantly increased after exposure to 1 μM of ACR, however *GRM8* expression was differentially decreased compared to undifferentiated cells during differentiation. Gene expression of *CACNA1E, CACNA1B, GRM1* and *GNG7* were significantly decreased after exposure to 70 μM of ACR and they were all differentially increased compared to undifferentiated cells during differentiation. *PLCD3* expression was significantly increased after exposure to 70 μM of ACR and was differentially increased compared to undifferentiated cells during differentiation. ACR significantly decreased the expression of *CREB1* after exposure to 1 μM and increased the expression after exposure to 70 μM. However, *CREB1* was not differentially expressed in untreated differentiated cells compared to undifferentiated SH-SY5Y cells but was investigated because of its central part of the CREB signalling pathway.

The protein level of total CREB increased concentration-dependently after exposure to 1 and 70 µM of ACR during 9 days of differentiation (Fig. 4b). A different pattern was observed when looking at the ratio of pCREB over total CREB. The ratio of pCREB/CREB decreased after exposure to 1 µM and increased after exposure to 70 µM of ACR (Fig. 4c). The ratio of pCREB was significantly different between the both ACR concentrations.

### Acrylamide interferes with important neuronal markers during differentiation

**Figure 5:**
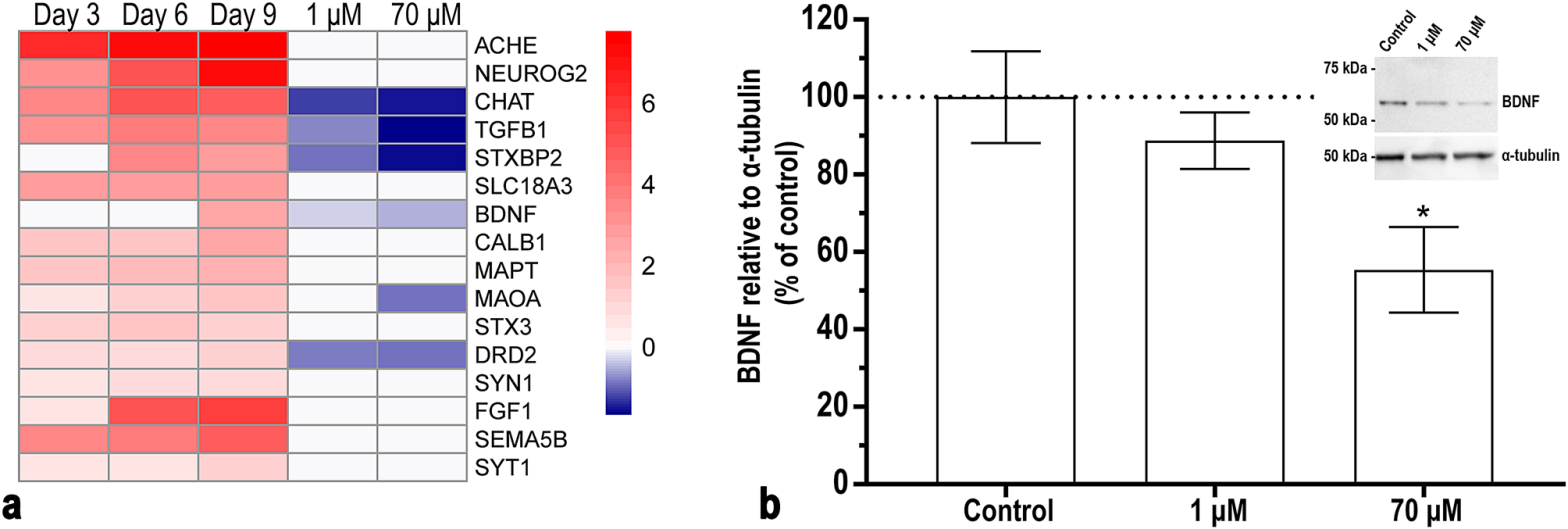
ACR interferes with genes that are important for neuronal differentiation during retinoic acid- induced SH-SY5Y cell differentiation.** (a)** Heatmap of the gene expression changes of several neuronal markers and other genes of interest (Day 3, Day 6 and Day 9 versus undifferentiated cells—RNA-sequencing input data) and following exposure to ACR (1 and 70 µM, after 9 days of differentiation versus untreated cells at the same time point—PCR input data). Colour coding refers to the log2(fold change). Genes are ranked according to decreasing log2(fold change) of the contrast Day 9 versus undifferentiated cells. **(b)** BDNF protein levels after 9 days of differentiation and exposure to 1 and 70 μM of ACR by western blot (image for one representative gel is included). The data from the protein levels were normalized against α-tubulin and presented as the mean ± SEM (N = 4). Results were analysed using one-way ANOVA followed by Dunnett’s multiple comparisons test. *p ≤ 0.05 compared to control (cells exposed to differentiation medium without ACR). Full length blots are presented in Supplementary Figure S4 online.

In addition to the markers studied in the RAR activation and CREB signalling pathways, we chose to include markers for cholinergic (*CHAT*) and dopaminergic (*DRD2* and *MAOA*) neurons due to their importance for neuronal differentiation in the SH-SY5Y cells. We also included synaptotagmin 1 (*SYT1*) that has been shown to form a protein adduct with ACR^15^, *MAOA* and *FGF1* that previously have been shown to be affected by ACR exposure^14,34^ and *BDNF* and tropomyosin receptor kinase B *(TrkB)* that are closely correlated with the CREB signalling pathway^25,35,36^. The expression was significantly decreased for 6 out of 16 genes after exposure to 70 μM of ACR and 5 of them were also decreased after exposure to 1 μM of ACR (Fig. 5a). The expression of *CHAT, TGFB1, STXBP2, BDNF* and *DRD2* were differentially increased in differentiated compared to undifferentiated cells but were all downregulated after exposure to ACR. *MAOA* was differentially increased compared to undifferentiated cells and was only significantly reduced after exposure to 70 μM of ACR. The effect of ACR on the protein level of BDNF followed the pattern of *BDNF* expression at the mRNA level, being significantly reduced after exposure with 70 µM (Fig. 5b).

## Discussion

We evaluated effects of ACR on RA-induced neuronal differentiation in the human neuroblastoma cell line SH-SY5Y. Concomitantly with our previous study on effects of ACR after 3 and 6 days of exposure during differentiation^28^, we here found that exposure with very low (picomolar) concentrations of ACR during 9 days attenuated differentiation by sustaining proliferation and reducing neurite outgrowth. Both cell proliferation and neurite outgrowth are hallmarks of neurodevelopment and are indicated as endpoints to be included in strategies for assessment of DNT using in vitro approaches^37^. Ogawa et al.^38^ showed that low doses of ACR in drinking water during gestation and weaning period decreased apoptosis of neuroblasts in hippocampus, suggesting a compensatory mechanism for impaired neurogenesis^38^. Increased proliferation due to attenuated differentiation can result in an imbalance in the number of neurons, connections between neurons and projections to different regions of the brain, a phenomenon that has been described in autism^39^. It has been shown that prefrontal cortex in children diagnosed with autism consists of up to 67% more neurons compared with brains of control children but the mechanisms for this observation are still unknown^40^. The causes of autism seem to be related to both genetic and environmental factors. Examples of environmental risk factors are prenatal exposure to certain medications such as valproate^41^ and antidepressant drugs^42^, especially during the first trimester of pregnancy. In utero exposure to valproate showed an eightfold increased risk for autism and changes the expression of genes that have been associated with autism in a mouse model^43^. Studying gene expression profiles in cells after exposure with chemicals during neuronal development and differentiation can provide mechanistic insight for underlying causalities for neurodevelopmental adversities^44^. In the case of autism, there are candidate risk genes encoding for calcium channels, calcium-regulated signalling proteins, BDNF, CREB, PI3K and ERK1/2^45^, i.e. some of the genes that were significantly affected by ACR in this study. This is a matter of concern since chemicals that interfere with these candidate genes might promote DNT.

Neuronal differentiation was induced by RA in this study. RA is an essential morphogen for nervous system development^46^ and has been shown to be important for neural differentiation, motoraxon outgrowth and neural patterning in many different in vitro model systems^47^. Herein, we observed that ACR decreased expression of key neuronal markers that were upregulated during RA-induced differentiation, i.e. *TGFB1, MAOA, DRD2* and *CHAT,* further indicating attenuated differentiation. RA is formed by a two-step oxidation of retinol (vitamin A). The first step is reversible where retinol is oxidized by retinol dehydrogenases (e.g. RDH10 and RDH16) to retinaldehyde^48^. The second step is an irreversible oxidation of retinaldehyde to RA by 3 different types of aldehyde dehydrogenases (ALDH1A1, ALDH1A2 and ALDH1A3)^49^. The reduction of retinaldehyde to retinol is performed by a short chain dehydrogenase reductase called DHRS3. RA can be catabolized by several members of the cytochrome p450 family (e.g. CYP26A1, CYP26B1, CYP26C1), a process important to prevent toxicity from retinol^50^. The genes for *RDH16, ALDH1A2, ALDH1A3, DHRS3* and *CYP26* were differentially expressed after 3, 6 and 9 days of differentiation. However, none of the genes were significantly affected after ACR exposure, indicating that ACR might not affect genes that are involved in the metabolism of RA per se. In the cytoplasm, RA is associated with the cellular retinoic acid-binding protein (CRABP), which translocates RA into the nucleus^51^. Inside the nucleus, RA can stimulate chromatin remodelling which influences gene expression of genes with specific cAMP response elements in their promoter regions^52^. Exposure to 1 μM and 70 μM of ACR significantly reduced the gene expression of *CRABP1* in differentiating SH-SY5Y cells, which might influence the translocation of RA to the nucleus. This in turn might result in reduced expression of RA-regulated genes since the absence of RA has been shown to induce the recruitment of histone deacetylase protein complexes that leads to histone H3 lysine 27 trimethylation and subsequent gene silencing^53^.

CREB is a key transcription factor involved in a wide array of critical cell functions such as survival, proliferation and differentiation^54^. CREB regulates genes that are inducible or constitutively expressed. Among these, a great number are related to neuronal function, such as genes coding for neurotransmitter synthesis and metabolism and neurotrophic factors like BDNF^35^. *Creb1* and cAMP responsive element modulator (*Crem*) knockout in mice caused a significant reduction in neuronal and glial precursor cells during CNS development^55^. Hence, alterations in CREB levels and activation status can lead to altered differentiation patterns in developing neurons^56^. Furthermore, there seems to be an important balance in gene expression that are regulated by CREB for normal brain development. An increase in CREB-mediated gene expression resulted in negative implications for the brain. It was shown in a transgenic mouse model that excessive CREB expression caused epileptic seizures and resulted in cell death via an excitotoxic mechanism rather than a pro-apoptotic mechanism as shown for CREB inactivation^57^. On the other hand, CREB has been shown to contribute to neuronal survival by regulating expression of antioxidant genes such as heme oxygenase-1, peroxisome proliferator-activated receptor gamma coactivator-1α and manganese superoxide dismutase^58–60^. Many chemicals that can be linked to DNT share a common pathophysiological cascade involving oxidative stress and impaired hippocampal neurogenesis. It has been suggested that the neuropathogenic processes might be accelerated by ACR since ACR can compete for the cysteine binding sites of nucleophilic scavengers^61^. Previous studies in rats have shown that ACR exposure increased the levels of reactive oxygen species (ROS) due to depletion of neural glutathione^62^. Hence, attenuated CREB-activated gene expression may play a role in ROS mediated neurotoxicity. The fact that ACR altered the expression of *CREB1* could possibly affect the ability of the cells to handle ACR induced toxicity related to increased levels of ROS^62^, resulting in an amplified toxicity. We observed an increase in the ratio of activated pCREB in cells exposed to 70 μM of ACR which might be explained by the fact that the concentration is just below the level of cytotoxicity for the SH-SY5Y cells and might indicate CREB-related expression of antioxidant related genes.

CREB can be activated via phosphorylation by different pathways, one of them is via BDNF binding to TrkB and the p75 neurotrophin receptor^63^. BDNF is one of the most well studied neurotrophins due to its complex roles in neuronal survival, neurogenesis and neuronal differentiation^64^. Vesicles with BDNF are located both pre- and postsynaptically and can be transported by retrograde and anterograde transport to different areas of the neurons^65^. BDNF can be secreted in its mature form (mBDNF) and in its pro-form (pro-BDNF), both of which are active signalling proteins^66^. The proteolytic cleavage of pro-BDNF as well as local translation and transport of BDNF is regulated by neuronal activity^65,67^. ACR has been found to inhibit kinesin-based fast axonal transport by reducing the affinity between the microtubules and kinesin through covalent modifications^68,69^. Hence, if axonal transport is affected, the BDNF delivery at the nerve terminals will be reduced, which might result in attenuated BDNF signalling and neuronal differentiation. Apart from the effect of ACR on microtubule-based transportation within the cells, our results showed that ACR also reduced the mRNA and protein levels of BDNF. Hence, ACR induced DNT might be partly associated with inhibition of axonal transport and delivery of existing BDNF to the axon terminals but also that there is less BDNF available for transport due to reduced protein and gene expression. BDNF has been shown to influence membrane excitability and synaptic transmission by its activation of the RAF-MEK-ERK pathway where the extracellular signal-regulated kinase (ERK) has been shown to phosphorylate synapsin^70^. In vitro studies have shown that BDNF increased the amount of neurotransmitter vesicles that were docked at the active zones in the synapses^71^. ACR has previously been shown to reduce neurotransmitter release by inhibiting the soluble NSF attachment protein receptor (SNARE) protein SNAP-25 and N-ethylmaleimide sensitive factor by adduct formation in vitro^72–74^. Here we observed that ACR decreased the expression of *STXBP2,* further supporting that ACR interferes with neurotransmitter release. Hence, ACR might reduce BDNF-CREB-mediated responses even further by inhibiting the positive feedback loop of the BDNF-CREB-mediated BDNF gene transcription and synaptic stability by reducing the release of BDNF both pre- and postsynaptically.

The effects observed after ACR exposure in our neurodevelopmental model system might have multiple implications. People are exposed to ACR throughout their lifetime, starting in utero. The average oral intake of ACR is estimated to 0.8–3 μg ACR/kg/day for adults and children are estimated to have a 2–3 times higher consumption when considering their food intake to body weight ratio^75^. Children are also at higher risk since they tend to eat more food that contains ACR^76^. There are few reports available on the actual free concentration of ACR in plasma of the general population. However, there is one study on documented ACR exposed workers showing that the unexposed control group had a plasma level of free ACR of around 1 μM^77^. Placental transfer of ACR has been measured to approximately 20%^5^, giving a rough estimation of foetal plasma concentration of approximately 0.2 μM. The fact that our results indicate that ACR altered signalling pathways at similar concentrations is a matter of concern since they seem to be physiologically relevant for foetal exposure. This might be particularly hazardous since adverse effects caused by ACR have been shown to be dose-dependent and accumulate over time in rats^78^.

In conclusion, our results reveal that ACR interferes with crucial signalling pathways and markers involved in neuronal differentiation in vitro and raise concern over the potential toxic outcomes in humans. Any chemical that alters the expression of CREB or BDNF signalling could have severe consequences since genes involved in these pathways have been linked to disorders such as autism^79,80^. Similarly, alterations to the RAR pathway have been previously linked to several developmental malformations, involving different organ systems including the nervous system, both in vitro^47^ and in vivo^81^. In addition, we showed that it is possible to identify enriched pathways during differentiation of SH-SY5Y cells and to study the impact of a neurotoxic chemical on these pathways. During the past decades there has been a shift towards using alternative methods to animal testing when it comes to toxicological testing of chemicals. The current DNT guidelines are based on animal studies (usually in rodents) (OECD TG 426)^82^, but effort is being put into developing robust and reliable alternatives involving the use of cell-based in vitro methods and computer modelling. The use of cell-based systems can be a valuable strategy in trying to elucidate the mechanism of action of a chemical. Furthermore, transcriptomic information may be useful in integrated approaches to testing and assessment (IATA) or risk assessment of chemical toxicity^83^. A cell model like the SH-SY5Y cell line, which is easy to handle, robust and relatively fast to differentiate, is an attractive tool for such studies, e.g. as a cheaper and less sophisticated alternative to human inducible pluripotent stem cell (hiPSC) models^37^. However, our results showing that concentrations of ACR that are relevant for human exposure may be harmful for developing neurons, should be validated in other, more complex models of the nervous system. hiPSCs may be the most relevant choice for that.

## Methods

### Chemicals and reagents

Acrylamide (99.9% purity) (A9099), resazurin (R7017), propidium iodide (P4170), Tween 20 (P1379), monoclonal anti-α-tubulin antibody mouse (T9026), Mouse IgG HRP Linked Whole Ab (GENA931) and Greiner CELLSTAR 96 well plates (M0562-32EA) were purchased from Sigma Aldrich (Sweden). Gibco phosphate-buffered saline (PBS), Gibco TrypLE Express Enzyme (1X) phenol red, Gibco minimum essential medium (MEM), Gibco MEM non-essential amino acid solution, Gibco Dulbecco’s modified Eagles medium: Nutrient mixture F-12 (DMEM/F-12), foetal calf serum (EU approved origin of South America, Ref:10270-106), Gibco L-glutamine, Gibco Pen-Strep (10,000 U/ml of penicillin and 10,000 μg/ml of streptomycin), Gibco N2 supplements, 100x (17502048), Goat anti-rabbit IgG (H + L) secondary antibody HRP (31460), Calcein-AM (C1430) and Hoechst (H3570) were purchased from Thermo Fisher Scientific (Sweden). All plastics used for cell culturing were from Corning Inc., (Corning NY) except for the CELLSTAR black cell culture microplate used for imaging mentioned above. RNA extraction kit RNeasy Plus Mini Kit was purchased from Qiagen. PrimePCR Positive Control SYBR Green Assay, PrimePCR DNA Contamination Control SYBR Green Assay, PrimePCR RNA Quality SYBR Green Assay, PrimePCR Reverse Transcription Control SYBR Green Assay, PrimePCR precasted 96-well plates, Precast TGX midi gels (5678033), Precision plus protein dual color standards (1610374), Bio-Rad Bradford protein determination assay (500-0006), Clarity Max Western ECL substrate (1705062), iScript cDNA synthesis kit and SsoAdvanced Universal SYBR Green Supermix were purchased from Bio-Rad (Sweden). Syringe filtration unit Filtropur S 0.2 (83.1826.001) were purchased from Sarstedt (Germany). CellTiter-Glo 2.0 (G9242) was purchased from Promega (Sweden). Syringe filtration unit Filtropur S 0.2 (83.1826.001) were purchased from Sarstedt (Germany). Western Blot -CREB primary antibody (ab32515), phosphorylated CREB s133 (ab194687), BDNF antibody (ab108319), 10X RIPA sample buffer (ab156034), protease and phosphatase inhibitor cocktail (ab201119) were purchased from Abcam (Sweden). Tris Base (97061-794), Glycine (101196X), methanol (BDH1135-4LP), NaCl (27,800.360) and centrifugal filters (polyethersulfone membranes) (516-0229) from VWR chemicals (Sweden). SENSE mRNA-Seq Library Prep Kit V2 (001.96) was purchased from Lexogen (Austria).

### Cell line and cell culturing

The human neuroblastoma SH-SY5Y cell line^84^ has been shown to be a very useful model system to study neuronal differentiation and function^85^. The SH-SY5Y cells develop into a neuronal-like phenotype upon differentiation with RA^86^. For routine cultures, the SH-SY5Y cells were seeded at a density of 27 × 10^3^ cells/cm^2^ in cell culture flasks. The cells were cultured as previously described by Attoff et al., 2016^28^ in MEM supplemented with 10% foetal calf serum, 1% non-essential amino acids, 2 mM L-glutamine, 100 μg streptomycin/mL, 100 U penicillin/mL. The confluent cells were detached once a week using TrypLE Express Enzyme and seeded in a new cell culture flask at the original density. For differentiation studies, the SH-SY5Y cells were seeded in routine culture medium (see individual experiment for density). Twenty-four hours after seeding the medium was changed to DMEM/F-12 medium supplemented with 1 mM L-glutamine, 100 U penicillin/mL, 100 μg streptomycin/mL, N2 supplements (1X) and 1 µM of RA. To avoid disrupting the neurites, half of the differentiation medium was removed and replaced with newly prepared medium every third day for the duration of the differentiation. The cells were kept in a humidified atmosphere of 5% CO_2_ in air at 37 °C.

### Culturing of cells and mRNA extraction

For the experimental setup, the SH-SY5Y cells were seeded at a density of 93.75 × 10^3^ cells/cm^2^ in 8 cm^2^ diameter cell culture dishes in routine culture medium. After the desired time of differentiation (i.e. 3, 6 or 9 days), the cells were harvested by detachment with TrypLE Express Enzyme and cell pellets were collected. Undifferentiated cells were harvested 24 h after seeding (at the start of differentiation for the differentiating cultures). The cell suspensions were centrifuged for 5 min at 500 g and the pellet was stored at − 80 °C until mRNA extraction. On the day of the experiment, the cells were lysed and mRNA was extracted using the Qiagen RNeasy Plus Mini Kit according to manufacturer’s instructions. mRNA concentration was determined by a Tecan Infinite M200 Pro (Tecan Trading AG) for the RNA-sequencing and by a NanoPhotometer P-class (Implen GmbH) for the Quantitative Reverse Transcription Polymerase Chain Reaction (RT-qPCR) analysis.

### RNA-sequencing and data analysis

Libraries for sequencing were prepared using the Lexogen SENSE mRNA-Seq Library Prep Kit V2 and were sequenced on Illumina HiSeq2000 in 100 bp paired-end (at an average of 6.4 million reads per sample). FastQC (version 0.10.1)^87^ was used to analyse the quality of the sequencing data and sequences were trimmed using Trimmomatic (version 0.33)^88^ to remove the bad quality sequences. Remaining reads were mapped to the human genome (Ensembl version 84) using Bowtie2 (version 2.2.6)^89^ and quantification of gene expression was achieved using RSEM (version 1.2.28)^90^. The gene expression normalization was done using DESeq2^91^ and the outliers were removed. The raw RNA-Seq data are deposited at European Nucleotide Archive (ENA) (accession number PRJEB23591).

Differential gene expression for the contrasts Day 3/Day 6/Day 9 vs. undifferentiated cells was assessed using Bioconductor DESeq2 (www.bioconductor.org)^91^ package running in R language version 3.4.1 (https://www.r-project.org). Principal component analysis plots of the rlog transformed count data were generated as part of the DESeq2 workflow. Genes with a false discovery rate (FDA) adjusted* p*-value ≤ 0.05 and absolute log2(fold change) > 1 were considered as differentially expressed. Venn diagrams of the DEGs between the three contrasts were generated using a web tool developed by the Bioinformatics & Evolutionary Genomics Laboratory at VIB/UGent, Belgium (https://bioinformatics.psb.ugent.be/webtools/Venn/). Canonical pathway analysis was performed on the DEGs using Ingenuity Pathway Analysis (IPA) software (license obtained from Ingenuity Systems, Redwood City, CA). The enriched pathways were narrowed down to the ones part of the ‘Neurotransmitters and other nervous system signaling’ and ‘Ingenuity toxicity pathways’ category. Heatmaps of the genes differentially expressed within the CREB and RAR activation pathways were generated using data output from IPA and the *pheatmap* Bioconductor package.

### Acrylamide exposure

ACR was dissolved in the cell culture medium used for differentiation. As previously described^28^ the ACR stock solution was sterile-filtered through a 0.2 µm polyethersulfone filter before added to the cells and then diluted to different concentrations ranging from 10^−21^-10^−3^ M depending on the assay. Cell cultures exposed to medium without ACR were used as a control. For all experiments, the cells were exposed to ACR 24 h after seeding, together with the change of the culture medium. ACR was added at every change of medium throughout the duration of the experiment. Fresh medium without ACR was added to control cells. A fresh stock solution of ACR we prepared immediately before addition to the cells for each exposure occasion.

### Number of cells

The SH-SY5Y cells were seeded in routine culture medium at a density of 12.5 × 10^3^ cells/cm^2^ in cell culture Petri dishes with a diameter of 6 cm. The cells were exposed to 1 and 70 μM of ACR for 9 days during differentiation as described above. The cells were detached using TrypLE Express Enzyme and counted in a Bürker chamber using a phase contrast microscope.

### Imaging cells and quantification of neurites

The number of neurites per cell and the total neurite outgrowth were studied after exposure to 1 μM and 70 μM of ACR compared to control. The SH-SY5Y cells were seeded in 6 cm diameter cell culture dishes at a density of 12.5 × 10^3^ cells/cm^2^. Each experiment was performed in technical duplicates, i.e. two wells per condition. Twenty-four hours after seeding, the medium was changed to differentiation medium. At the same time, 1 μM, 70 μM or no ACR was added to the cells. The medium was changed every third day. After 9 days of differentiation and exposure, the cells were photographed using a phase contrast microscope (Olympus). Microscopy images were captured at 150 × magnification using a SC50 5-megapixel colour camera (Olympus SC50). Three images of each technical duplicate were depicted and analysed using the Fiji package for ImageJ, where the number of cells in each image was calculated using the “Cell counter” plugin. The neurite outgrowth analysis was estimated using the “NeuronJ” plugin where all neurites were manually traced and measured. Cell bodies and neurites that were longer than the diameter of the cell body were counted and the number of neurites per cell was estimated as previously described^92^. Three pictures were taken for each technical replicate and the mean value for each situation was calculated. The mean value was considered as one N. The statistical analyses for this experiment was based on N = 3, i.e. three individual experiments, each performed in duplicates that were depicted in triplicates.

### Quantitative reverse transcription polymerase chain reaction

The effect of 1 μM and 70 μM ACR exposure on the selected genes involved in RAR activation and CREB signalling were analysed using RT-qPCR. For the experimental setup, the SH-SY5Y cells were seeded at a density of 12.5 × 10^3^ cells/cm^2^ in 10 cm diameter cell culture dishes in routine culture medium. After 9 days of differentiation and ACR exposure the cells were harvested and mRNA was extracted in the same way as for the RNA-sequencing analysis. As previously described^93^, pre-casted white PrimePCR plates were designed by and purchased from Bio-Rad. Two μg of each RNA sample were reverse transcribed into cDNA using iScript cDNA Synthesis Kit from Bio-Rad. Real-time qPCR reactions were carried out using 10 ng of cDNA per reaction as described by Bio-Rad in the PrimePCR instruction manual, including experimental control assays for reverse transcription (PrimePCR Reverse Transcription Control SYBR Green Assay), genomic DNA (PrimePCR DNA Contamination Control SYBR Green Assay), RNA quality (PrimePCR RNA Quality SYBR Green Assay) and PCR performance (PrimePCR Positive Control SYBR Green Assay). The reaction was performed in a CFX96 Touch Real-Time PCR Detection System (Bio-Rad) using SsoAdvanced Universal SYBR Green Supermix. The data were analysed using the Bio-Rad CFX manager 3.1 software system. The samples were normalized against 3 reference genes: albumin (Alb), heat shock protein 90ab (Hsp90ab1) and ribosomal protein large P1 (Rplp1) which were identified to be equally expressed during all stages of differentiation relative to the total amount of mRNA in the whole genome RNA expression sequencing analysis^94^.

### Western blot

In order to determine the protein levels of BDNF, CREB and Ser133 pCREB, SDS-polyacrylamide gel electrophoresis was used to separate the proteins according to molecular weight and further detect them with western blot using specific primary antibodies and horse radish-conjugated secondary antibodies. SH-SY5Y cells were seeded in 10 cm diameter cell culture dishes at a density of 12.5 × 10^3^ cells/cm^2^. After 24 h, the medium was changed to differentiation medium and at the same time the cells were exposed to ACR at a concentration of 0, 1 or 70 µM. The medium with or without ACR was changed every 3 days. At exposure day 9, the cells were first washed with ice cold phosphate buffer saline (PBS). The PBS washing solution was collected into a 2 ml Eppendorf tube and centrifuged for 15 min at 4 °C and 12,000 rpm to collect cells that might detach during the washing step. The cells remaining in the plates were lysed in 500 µl radioimmunoprecipitation assay (RIPA) buffer containing protease and phosphatase inhibitors for 30 min on ice. The lysed cells were scraped gently, and the suspension was collected in the Eppendorf tube from the previous step and centrifuged for 20 min at 4 °C and 12,000 rpm. Finally, the supernatant was collected for protein concentration determination and the pellet was discarded. The protein concentration was determined by the Bradford assay according to the manufacturer’s instructions and once determined the cell lysates were aliquoted and boiled at 95 °C in 6 × Laemmli buffer for 5 min. Thirty µg of each protein lysate were separated in a 10% SDS-polyacrylamide gel at 100 V. The proteins were transferred to nitrocellulose membranes at 70 mA at 4 °C for 60 min. The membrane was cut in two parts guided by ponceau red staining and then incubated overnight at 4 °C with rabbit anti-CREB (1:1000), rabbit anti-pCREB (1:500) or rabbit anti-BDNF (1:500). The membranes were washed in tris buffered saline containing tween-20 (TBST) and then incubated with secondary antibody horseradish peroxidase-conjugated anti-rabbit IgG (1:50,000) for 60 min at room temperature followed by an incubation with ECL substrate. The bands were visualized using ChemiDoc XRS + image system (Bio-Rad). After visualization, the membranes were washed in TBST and then incubated overnight at 4 °C with mouse anti-α-tubulin (1:3000) which was used as a loading control. The membranes were washed in TBST and then incubated with secondary antibody horseradish peroxidase-conjugated anti-mouse IgG (1:50 000) for 60 min at room temperature. The visualization process followed the same procedure as described above. The relative luminescence intensity for each band was analysed by using the Image Lab 3.0 software program (Bio-Rad).

### Statistical analyses

GraphPad Prism 7.02 was used for statistical analysis of the data. See individual experiment for further details.

## Supplementary information

Supplementary file1

## Abbreviations

AC: Adenylyl cyclase
ACR: Acrylamide
ALDH: Retinaldehyde dehydrogenase
BDNF: Brain-derived neurotrophic factor
CACN: Voltage-dependent calcium channel
CNS: Central nervous system
CREB: cAMP response element-binding protein
DEG: Differentially expressed genes
DNT: Developmental neurotoxicity
FDR: False discovery rate
hiPSC: Human inducible pluripotent stem cells
IATA: Integrated approach for testing assessment
mGLUR: Metabotropic glutamate receptor
NS: Nervous system
RA: All-trans retinoic acid
iGLUR: Ionotropic glutamate receptor
IPA: Ingenuity pathway analysis
pCREB: Phosphorylated CREB
PI: Propidium iodide
PI3K: Phosphoinositide 3-kinase
PKC: Protein kinase C
PLC: Phospholipase C
RAR: Retinoic acid receptor
RBP: Retinol-binding protein
RDH: Retinol dehydrogenase
ROS: Reactive oxygen species
RT-qPCR: Quantitative reverse transcription polymerase chain reaction
SWI-SNF: SWItch/sucrose non-fermentable
TGF-β: Transforming growth factor beta
TrkB: Tropomyosin receptor kinase B receptor
