# Supplementary material for: Acrylamide alters CREB and retinoic acid signalling pathways during differentiation of the human neuroblastoma SH-SY5Y cell line
Source: Sci Rep. 2020 Oct 7;10:16714. doi: 10.1038/s41598-020-73698-6 (PMC7541504; doi:10.1038/s41598-020-73698-6)
Supplement: Supplementary file 1 — Supplementary file1 [file 41598_2020_73698_MOESM1_ESM.pdf]

Acrylamide alters CREB and retinoic acid signalling pathways during differentiation of the human neuroblastoma SH-SY5Y cell line

Kristina Attoff[a], Ylva Johansson[a], Andrea Cediel Ulloa[b,c], Jessica Lundqvist[a,c], Rajinder Gupta[d], Florian Caiment[d], Anda Gliga[b], Anna Forsby[a,c*]

a) Department of Biochemistry and Biophysics, Stockholm University, Stockholm, Sweden
b) Institute of Environmental Medicine, Karolinska Institutet, Stockholm, Sweden
c) Swedish Toxicology Sciences Research Center (Swetox), Unit of Toxicology Sciences, Karolinska Institutet, Södertälje, Sweden
d) Department of Toxicogenomics, School of Oncology and Developmental Biology (GROW), Maastricht University, Maastricht, The Netherlands

* Corresponding author
Email: anna.forsby@dbb.su.se

Supplementary information

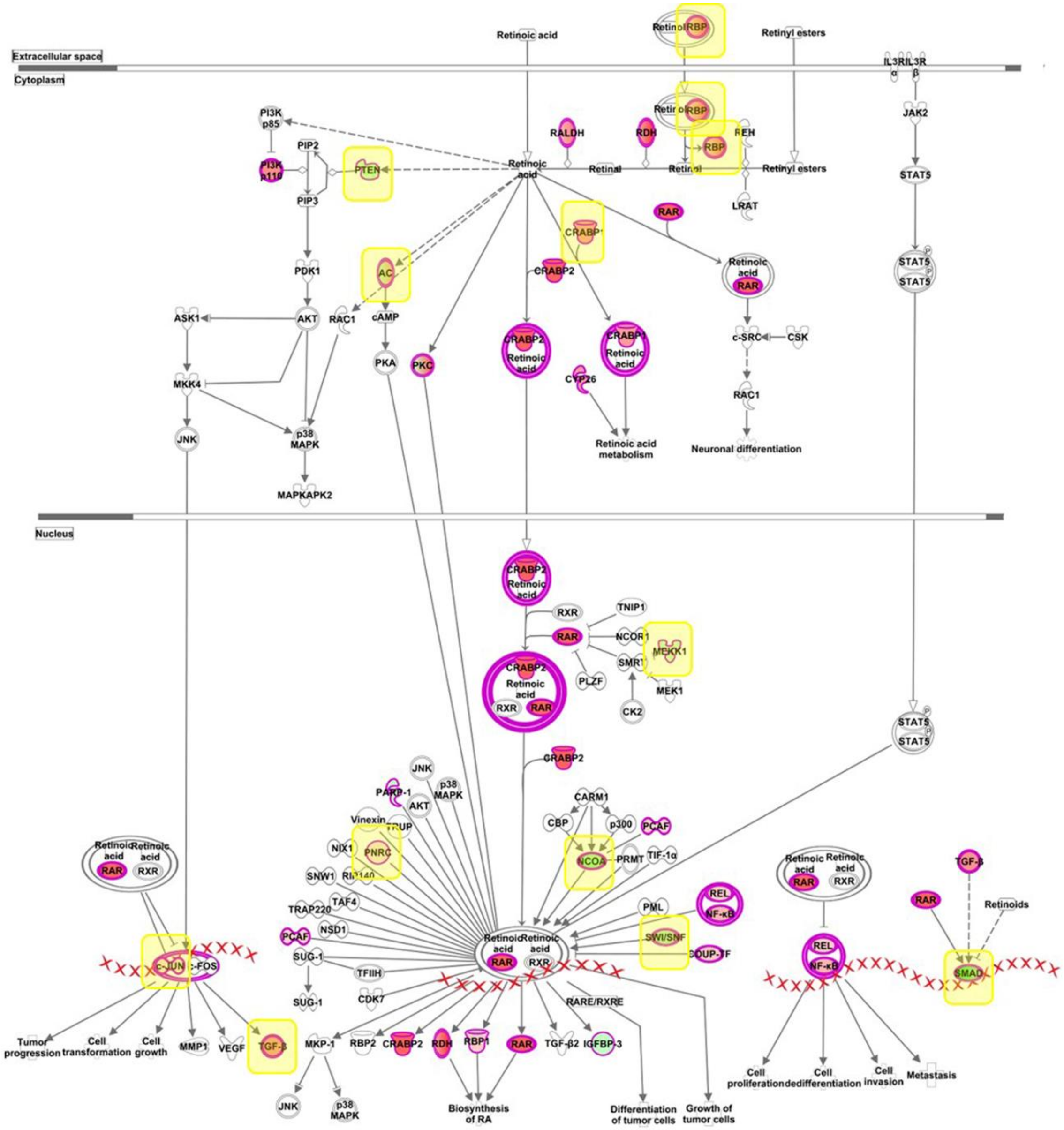

Figure S1. ACR interferes with the RA receptor signalling pathway during differentiation of SH-SY5Y cells. The 'RAR signalling pathway' is enriched after 9 days of differentiation in SH-SY5Y cells. Colour code for gene expression changes: red – upregulation, green – downregulation, red/green gradient – molecules part of this complex have their gene expression both up- and downregulated, yellow shading indicates molecules that have their gene expression altered after ACR exposure. The map of signalling pathway is obtained from the Ingenuity Pathway Analysis (IPA) software.

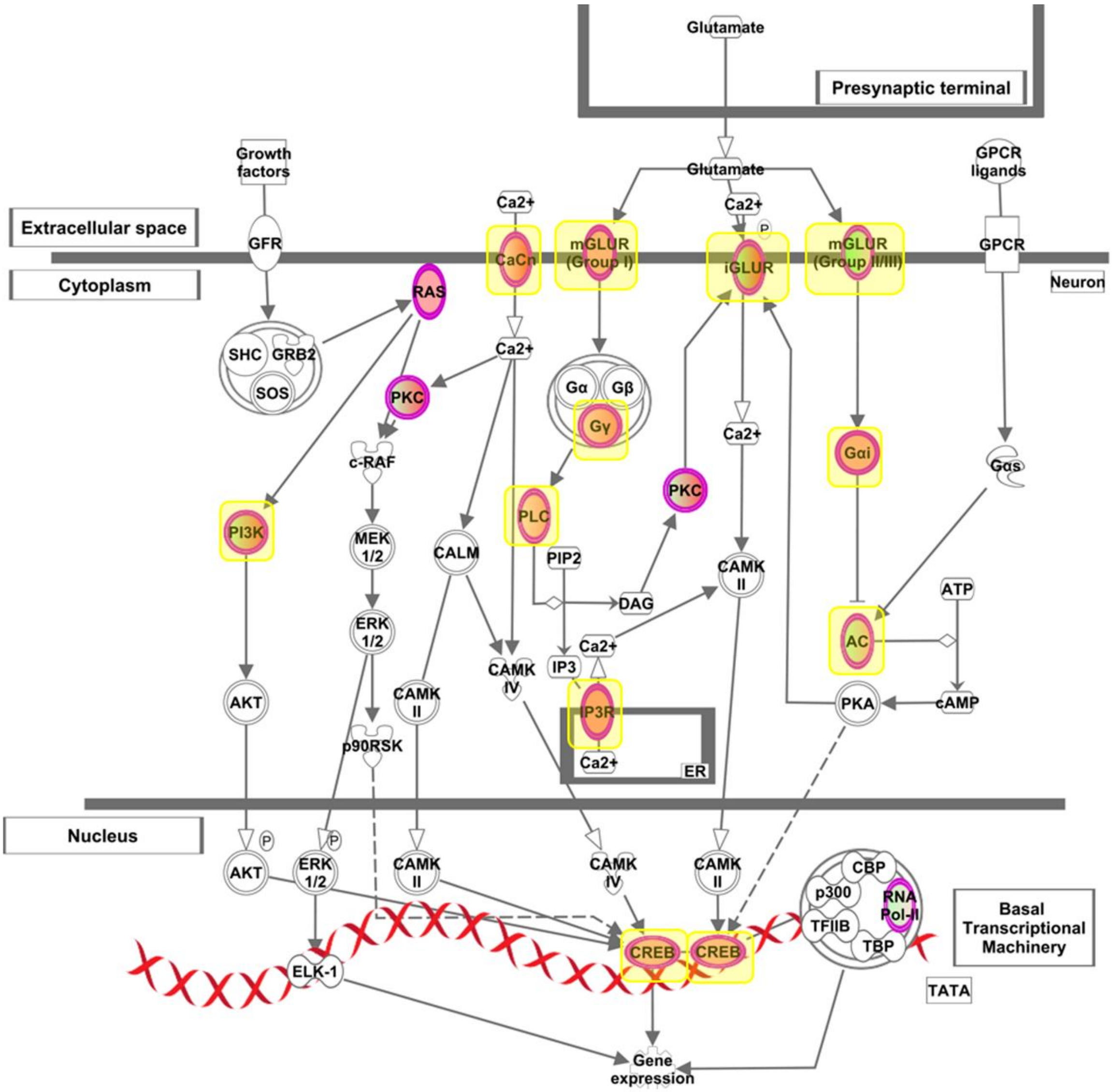

Figure S2. ACR interferes with the CREB signalling pathway during differentiation of SH-SY5Y cells. The 'CREB signalling pathway in neurons' is enriched after 9 days of differentiation in SH-SY5Y cells. Colour code for gene expression changes: red – upregulation, green – downregulation, red/green gradient – molecules part of this complex has their expression both up- and downregulated, yellow shading indicates molecules that have their expression altered after ACR (70 µM) exposure. The map of signalling pathway is obtained from the Ingenuity Pathway Analysis (IPA) software. Colour coding refers to the log2(fold change). Genes are ranked according to decreasing log2(fold change) of the contrast Day 9 versus undifferentiated cells.

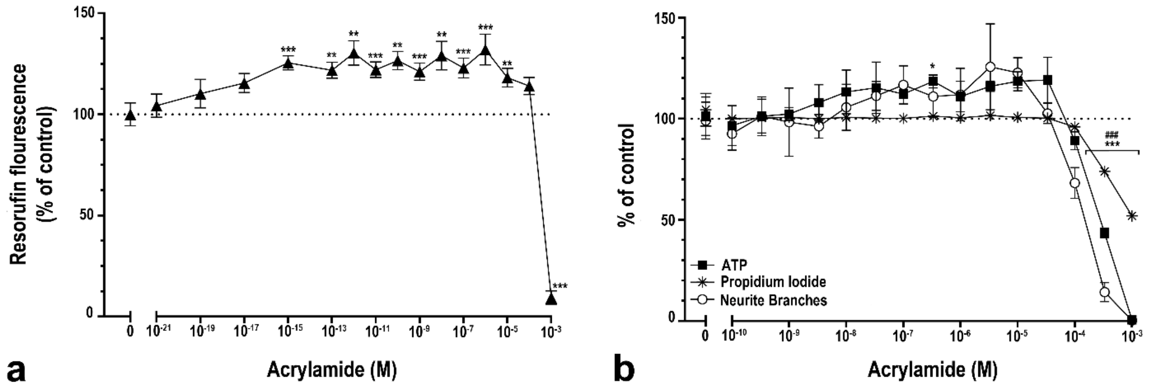

Figure S3. Effects of ACR on viability, amount of viable cells and neurite branching during differentiation of SH-SY5Y cells. a) Resazurin reduction during 9 days of differentiation and ACR exposure. Briefly, Resazurin dye has been broadly used to measure cell viability in cytotoxicity assays and its low toxicity compared to the MTT assay has made it a more suitable choice when studying cell viability. The method is based on enzymatic reduction of resazurin by viable cells to the highly fluorescent pink reagent resorufin. A 20X stock solution was prepared by dissolving resazurin sodium salt in 0.1 M NaOH and PBS. The solution was filter sterilized and stored protected from light at 4°C. For all experiments, the cells were seeded in clear 96-well plates at a density of 12.5 x 10^3 cells/cm^2. The cells were exposed for 9 days to ACR diluted in differentiation medium in concentrations ranging from 10^{-21} to 10^{-3} M. The medium was changed every third day, where 75 μL of medium without or with ACR was removed and 75 μL of fresh medium without or with ACR in the same concentrations was added to each well. After 9 days of exposure, 50 uL of cell culture medium was removed from each well and subsequently, 50 μL of the resazurin solution (2X dissolved in DMEM:F12 medium) was added and the plate was incubated for 2 hours at 37°C. Resorufin fluorescence was measured from the top at excitation 540 nm and at emission 590 nm with a FlexStation II fluorometer (Molecular Devices). **b)** Multiplex neurite branching-PI-ATP assay during 9 days of differentiation and ACR exposure. High content imaging was used to measure the neurite branching by using the cell permeant dye calcein-AM. Calcein-AM is hydrolysed after entering the cell, producing a fluorescent signal which allows the visualization of the cellular cytoplasm of viable cells. The correlation between cell bodies and nuclei was determined by using Hoechst as nuclear staining. Distinction between viable and dead cells was achieved with the dye propidium iodide (PI). PI is membrane impermeable for viable cells but can pass through the leaky membrane of damaged/dead cells where it binds to double stranded DNA by intercalating between base pairs. Here, the results are visualised as PI-resistant cells, i.e. number of PI-unstained cells in relation to total number of cells, expressed as % of unexposed control cells. For experiments, the SH-SY5Y cells were seeded at a density of 12.5 x 10^3 cells/cm^2 in black microclear bottom 96-well plates in routine culture medium. After 24 hours the medium was changed to differentiation medium and at the same time the cells were exposed to ACR in a range from 10^{-3} to 10^{-10} M. Three technical replicates were included for each concentration. Every third day, half of the medium was replaced with freshly prepared ACR dilutions and differentiation medium without ACR was added to the negative controls. The cells were imaged after 9 days of differentiation with an automated imaging system (ImageXpress Micro, Molecular Devices) and image acquisition software MetaXpress (Molecular Devices) using a modified version of the neurite outgrowth application module. Before imaging, the cells were incubated for 20 minutes at 37°C with a dye solution containing 1 μM calcein-acetoxymethylester, 5 μg/mL Hoechst and 5 μM PI. Following incubation, 130 μL of cell medium was added to all wells after which the image acquisition started. In each well, 4 sites were imaged under a 10x objective lens. Different wavelengths were used for detection of the different dyes, i.e. Hoechst (excitation 377/50 nm, emission 447/60 nm and 100 ms exposure time), calcein (excitation 482/35 nm, emission 536/40 nm and 120 ms exposure time) and PI (excitation 562/40 nm, emission 624/40 nm and 400 ms exposure time). After imaging, the ATP content of the cells was measured with CellTiter-Glo 2.0 (Promega). This assay uses a thermostable luciferase which produces a luminescent signal proportional to the amount of ATP present in the analysed samples. Cell lysis was induced in a plate shaker with the CellTiter-Glo 2.0 reagent for 2 minutes followed by a 15-minute incubation of the samples at room temperature. Luminescence was recorded for 300 ms by a plate reader (Tecan Infinite M200 Pro and the Tecan i-control 3.7.3.0 software). Results were analysed using one-way ANOVA followed by Dunnett's multiple comparisons test or Fisher's LSD test. Bars represent the mean ± SEM. *$p \leq 0.05$, **$p \leq 0.01$, ***$p \leq 0.001$ compared to control (cells exposed to medium without acrylamide). ###$p \leq 0.001$ between PI-resistance and neurite branches.

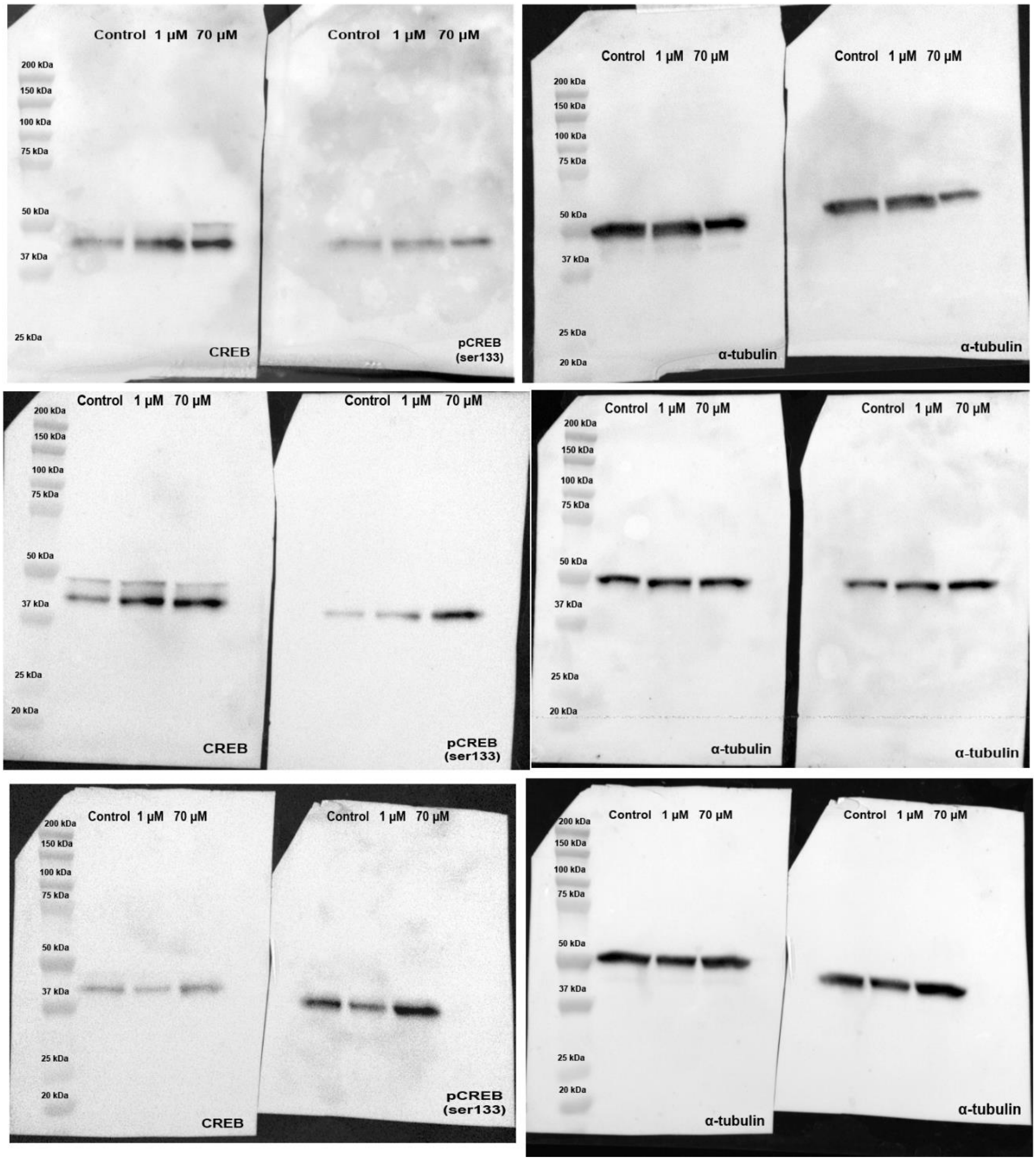

Figure S4. Full length blots of Figure 4b and 4c. CREB, pCREB and α-tubulin protein levels were measured in SH-SY5Y cells exposed to ACR during 9 days of differentiation at three different occasions (N=3). Methodological details are described in the main text.

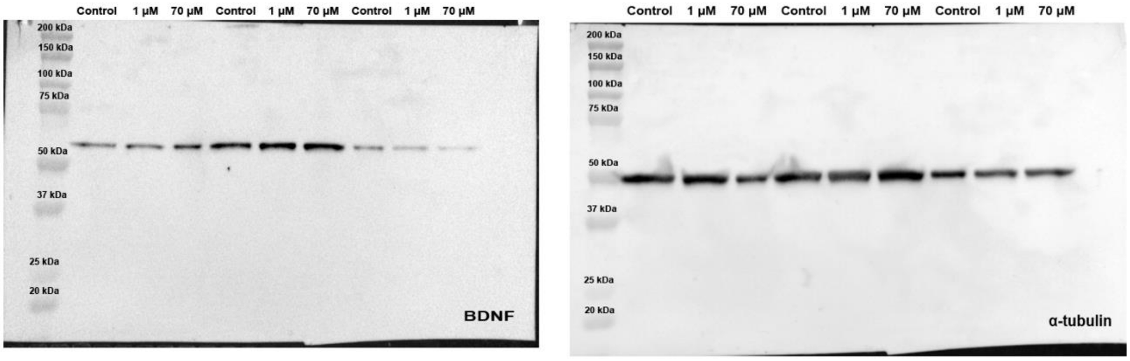

Figure S5. Full length blots of Figure 5b. BDNF (left) and α-tubulin (right) protein levels were measured in SH-SY5Y cells exposed to ACR during 9 days of differentiation at three different occasions (N=3). Methodological details are described in the main text.

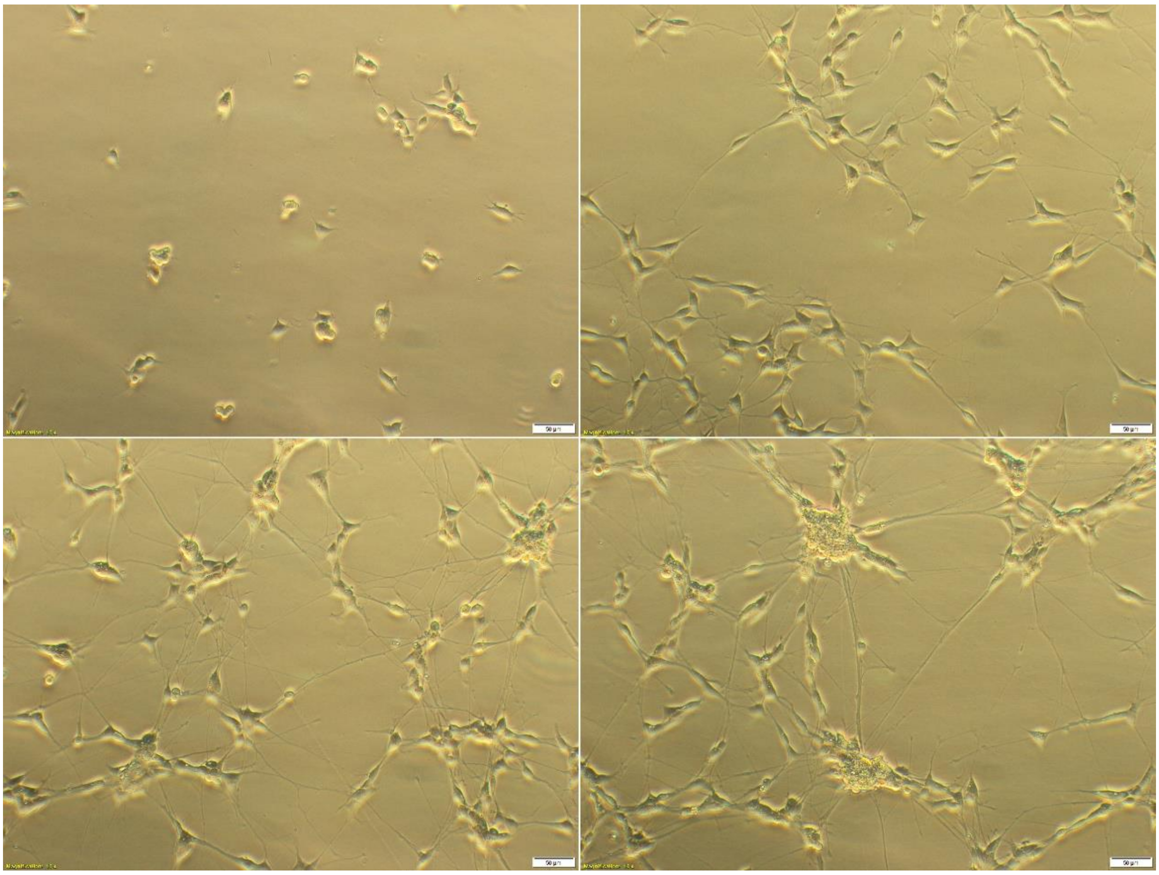

Figure S6. Unprocessed version of Figure 1a-d. SH-SY5Y cells imaged throughout RA-induced differentiation. Differentiating cells were depicted under the Cells were depicted under a Olympus phase contrast microscope at 150x magnification using a SC50 5-megapixel colour camera (Olympus SC50) at day 0 (upper left), 3 (upper right), 6 (lower left) and 9 (lower right). Scale bar = 50 µm.

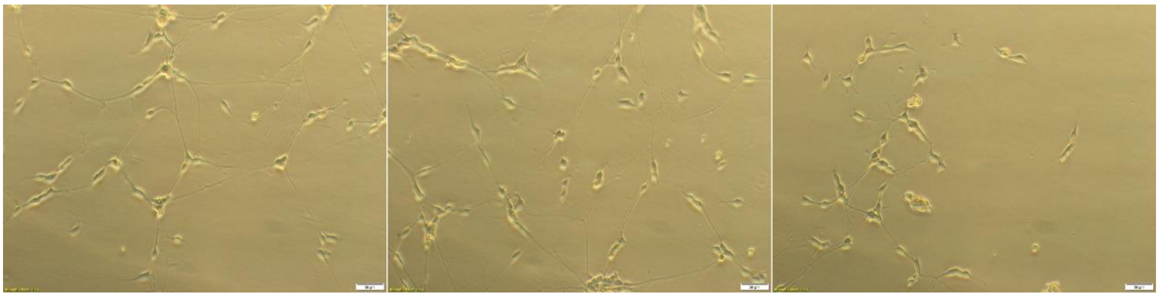

Figure S7. Unprocessed version of Figure 2a-c. SH-SY5Y cells imaged after exposure with ACR during 9 days of RA-induced differentiation. The cells were exposed to 0 µM (left), 1 µM (middle) and 70 µM (right) ACR. Cells were depicted under a Olympus phase contrast microscope at 150x magnification using a SC50 5-megapixel colour camera (Olympus SC50). Scale bar = 50 µm

Table S1. Genes included in the real time quantitative RT-PCR (qPCR). The differentially expressed genes (DEGs) in the RAR activation and CREB signaling pathways together with other relevant genes for acrylamide exposure and neuronal development were selected from RNAseq transcriptomic analysis based on Log2(fold change) values >1 (upregulation) or <-1 (downregulation), padj <0.05, indicated after 9 days of retinoic acid-induced differentiation in SH-SY5Y cells. DEGs occurring in other enriched pathways are indicated.

Genes in the RAR activation pathway*	Log2 fold change (green up, red down)	Gene description	Axonal guidance	Dopamine-DARPP32 feedback in cAMP Signaling	GABA Receptor Signaling	Glutamate receptor signalling	Differentia-tion of neurons	Developme nt of neurons	Affected by ACR in qPCR
DHRS3	10,64	dehydrogenase/reductase 3(DHRS3)							
CRABP2	5,11	cellular retinoic acid binding protein 2(CRABP2)							
PRKCG	5,50	protein kinase C gamma(PRKCG)	X	X					
CYP26A1	3,13	cytochrome P450 family 26 subfamily A member 1(CYP26A1)							
RARB	5,01	retinoic acid receptor beta(RARB)					X		
TGFB1	3,54	transforming growth factor beta 1(TGFB1)					X	X	DOWN
PIK3CD	3,64	phosphatidylinositol-4,5-bisphosphate 3-kinase catalytic subunit delta(PIK3CD)	X					X	
ALDH1A2	4,18	aldehyde dehydrogenase 1 family member A2(ALDH1A2)					X		
ADCY5	3,13	adenylate cyclase 5(ADCY5)		X	X				DOWN
RBP7	3,98	retinol binding protein 7(RBP7)							DOWN
CRABP1	3,24	cellular retinoic acid binding protein 1(CRABP1)							DOWN
ALDH1A3	2,42	aldehyde dehydrogenase 1 family member A3(ALDH1A3)							
RBP3	4,72	retinol binding protein 3(RBP3)							
NFKB2	2,37	nuclear factor kappa B subunit 2(NFKB2)							
ADCY4	3,14	adenylate cyclase 4(ADCY4)		X	X				

RDH16	2,97	retinol dehydrogenase 16 (all-trans)(RDH16)							
RBP1	1,61	retinol binding protein 1(RBP1)							
KAT2B/PCAF	1,53	lysine acetyltransferase 2B/histone acetyltransferase PCAF							DOWN
NR2F1	1,71	nuclear receptor subfamily 2 group F member 1(NR2F1)						X	
RELB	1,63	RELB proto-oncogene, NF-kB subunit(RELB)						X	
PRKCD	1,41	protein kinase C delta(PRKCD)	X	X					
JUN	1,31	Jun proto-oncogene, AP-1 transcription factor subunit(JUN)						X	DOWN
PNRC1	1,05	proline rich nuclear receptor coactivator 1(PNRC1)							DOWN
PARP-1	-1,13	poly(ADP-ribose) polymerase 1(PARP1)							
SMAD9	-1,25	SMAD family member 9(SMAD9)							DOWN/UP
ADCY8	-1,53	adenylate cyclase 8(ADCY8)		X	X				DOWN
SMARCA4	-1,03	SWI/SNF related, matrix associated, actin dependent regulator of chromatin, subfamily a, member 4(SMARCA4)							
MAP3KI/MEKK1	-1,14	Mitogen-Activated Protein Kinase Kinase Kinase 1							UP
**PRKCA**	-1,06	protein kinase C alpha(PRKCA)	X	X			X	X	
PBRM1	-1,27	polybromo 1(PBRM1)							DOWN
PTEN	-1,38	phosphatase and tensin homolog(PTEN)					X	X	DOWN
SMAD7	-1,58	SMAD family member 7(SMAD7)							
DPF1	-1,77	double PHD fingers 1(DPF1)							
IGFBP-3	-1,18	insulin like growth factor binding protein 3(IGFBP3)							

CITED2	-2,02	Cbp/p300 interacting transactivator with Glu/Asp rich carboxy-terminal domain 2(CITED2)							DOWN
SMAD6	-5,00	SMAD family member 6(SMAD6)							
**Genes in the CREB Signaling in Neurons network***									
GRIK4	6,55	glutamate ionotropic receptor kainate type subunit 4(GRIK4)				X			
CACNG1	6,36	calcium voltage-gated channel auxiliary subunit gamma 1(CACNG1)			X				DOWN
PRKCG	5,50	protein kinase C gamma(PRKCG)	X	X					
TLR9	5,62	toll like receptor 9(TLR9)	X						
CACNA1E	5,55	calcium voltage-gated channel subunit alpha1 E(CACNA1E)		X	X				DOWN
GNG3	4,46	G protein subunit gamma 3(GNG3)	X						
ITPR3	3,88	inositol 1,4,5-trisphosphate receptor type 3(ITPR3)		X					DOWN
PIK3CD	3,64	phosphatidylinositol-4,5-bisphosphate 3-kinase catalytic subunit delta(PIK3CD)	X	X				X	
PLCH1	3,85	phospholipase C eta 1(PLCH1)	X	X					
OPN1SW	3,32	opsin 1 (cone pigments), short-wave-sensitive(OPN1SW)	X	X					DOWN
GRIN2D	3,21	glutamate ionotropic receptor NMDA type subunit 2D(GRIN2D)		X		X			
CACNA1G	2,82	calcium voltage-gated channel subunit alpha1 G(CACNA1G)			X				
ADCY5	3,13	adenylate cyclase 5(ADCY5)		X	X				DOWN
GRM1	3,18	glutamate metabotropic receptor 1(GRM1)				X		X	DOWN

RRAS	2,89	related RAS viral (r-ras) oncogene homolog(RRAS)							
CREB5	3,08	cAMP responsive element binding protein 5(CREB5)		X					DOWN
PLCD1	2,56	phospholipase C delta 1(PLCD1)	X	X					
CACNA2D2	2,52	calcium voltage-gated channel auxiliary subunit alpha2delta 2(CACNA2D2)			X				
GNG2	1,90	G protein subunit gamma 2(GNG2)	X			X			
FGFR4	2,06	fibroblast growth factor receptor 4(FGFR4)	X						DOWN
GNG7	2,01	G protein subunit gamma 7(GNG7)	X			X			DOWN
ADCY4	3,14	adenylate cyclase 4(ADCY4)		X	X				
CREB3L3	2,71	cAMP responsive element binding protein 3 like 3(CREB3L3)		X					
CACNG5	1,43	calcium voltage-gated channel auxiliary subunit gamma 5(CACNG5)			X				
PLCB2	1,63	phospholipase C beta 2(PLCB2)	X						
GRIN2C	1,44	glutamate ionotropic receptor NMDA type subunit 2C(GRIN2C)				X			
PLCD3	1,46	phospholipase C delta 3(PLCD3)	X						UP
GRM7	1,51	glutamate metabotropic receptor 7(GRM7)				X			DOWN
CACNG4	1,29	calcium voltage-gated channel auxiliary subunit gamma 4(CACNG4)			X				
GRM4	1,82	glutamate metabotropic receptor 4(GRM4)				X		X	
PLCH2	1,64	phospholipase C eta 2(PLCH2)	X	X					DOWN
NOTUM	1,83	NOTUM, palmitoleoyl-protein carboxylesterase(NOTUM)	X	X					
CACNA1B	1,52	calcium voltage-gated channel subunit alpha1 B(CACNA1B)			X				DOWN
PRKCD	1,41	protein kinase C delta(PRKCD)	X	X					

CACNG7	1,32	calcium voltage-gated channel auxiliary subunit gamma 7(CACNG7)			X				
CACNA1D	1,45	calcium voltage-gated channel subunit alpha1 D(CACNA1D)		X	X				
FGFR1	1,23	fibroblast growth factor receptor 1(FGFR1)	X					X	
POLR2J2	1,15	RNA polymerase II subunit J2(POLR2J2)							
ADCY8	-1,53	adenylate cyclase 8(ADCY8)		X	X				DOWN
**PRKCA**	-1,06	protein kinase C alpha(PRKCA)	X	X			X	X	
GRIA2	-1,36	glutamate ionotropic receptor AMPA type subunit 2(GRIA2)				X			DOWN
FGFR2	-1,40	fibroblast growth factor receptor 2(FGFR2)	X				X	X	DOWN
GRM2	-1,36	glutamate metabotropic receptor 2(GRM2)				X			
IRS1	-1,43	insulin receptor substrate 1(IRS1)	X						
POLR2F	-1,31	RNA polymerase II subunit F(POLR2F)							
GRID1	-2,05	glutamate ionotropic receptor delta type subunit 1(GRID1)				X			
GRM8	-2,86	glutamate metabotropic receptor 8(GRM8)				X			DOWN
**From BDNF-TrkB pathway**									
BDNF	2,61	Brain Derived Neurotrophic Factor	X				X	X	DOWN
**Protein form adducts with ACR (Barber et al. https://doi.org/10.1093/toxsci/kfm210)**									
SYT1	1,18	Synaptotagmin							
**ACR and DNT from literature**									

FGF1 (Chen et al., Arch Toxicol. 2014 Mar;88(3):769-80.)	5,84	Fibroblast Growth Factor 1					X		
MAOA (Pan et al., Toxicol Lett. 2015 Jul 2;236(1):60-8.)	1,43	Monoamine Oxidase A							DOWN
SEMA5B (Attoff et al., PLoS One 12, e0190066 (2017))	4,61	Semaphorin 5B							
MAPT (Attoff et al., PLoS One 12, e0190066 (2017))	2,11	Microtubuli associated protein Tau					X	X	
**Neuronal biomarkers**									
SYN1	1,08	Synapsin 1						X	
CALB1	2,40	Calbindin 1							
STXBP2	3,01	Syntaxin Binding Protein 2							DOWN
TGFB1	3,54	Transforming Growth Factor Beta 1					X	X	DOWN
SLC18A3								X	
STX3	1,39	Syntaxin 3						X	DOWN
**Dopamigic markers**									
DRD2	1,30	Dopamine receptor D2		X				X	DOWN
NEUROG2	7,19	Neurogenin 2					X	X	

**Cholinergic markers**									DOWN
ChAT	4,90	Choline O-Acetyltransferase					X	X	DOWN
AChE	7,77	Acetylcholinesterase						X	
SLC18A3 (VAChT)	2,98	Solute Carrier Family 18 Member A3							

*) Duplicates in matching colour coding
